# Abundance of montane salamanders over an elevational gradient

**DOI:** 10.1002/ece3.7142

**Published:** 2020-12-29

**Authors:** Daniel J. Hocking, John A. Crawford, William E. Peterman, Joseph R. Milanovich

**Affiliations:** ^1^ Biology Department Frostburg State University Frostburg MD USA; ^2^ National Great Rivers Research and Education Center East Alton IL USA; ^3^ School of Environment and Natural Resources The Ohio State University Columbus OH USA; ^4^ Department of Biology Loyola University Chicago Chicago IL USA

**Keywords:** amphibian, climate change, Great Smoky Mountains National Park, *N*‐mixture model, Plethodontidae

## Abstract

Climate change is expected to systematically alter the distribution and population dynamics of species around the world. The effects are expected to be particularly strong at high latitudes and elevations, and for ectothermic species with small ranges and limited movement potential, such as salamanders in the southern Appalachian Mountains. In this study, we sought to establish baseline abundance estimates for plethodontid salamanders (family: Plethodontidae) over an elevational gradient in Great Smoky Mountains National Park. In addition to generating these baseline data for multiple species, we describe methods for surveying salamanders that allow for meaningful comparisons over time by separating observation and ecological processes generating the data. We found that *Plethodon jordani* had a mid‐elevation peak (1,500 m) in abundance and *Desmognathus wrighti* increased in abundance with elevation up to the highest areas of the park (2025 m), whereas *Eurycea wilderae* increased in abundance up to 1,600 m and then plateaued with increasing uncertainty. Litter depth, herbaceous ground cover, and proximity to stream were also important predictors of abundance (dependent upon species), whereas daily temperature, precipitation, ground cover, and humidity influenced detection rates. Our data provide some of the first minimally biased information for future studies to assess changes in the abundance and distribution of salamanders in this region. Understanding abundance patterns along with detailed baseline distributions will be critical for comparisons with future surveys to understand the population and community‐level effects of climate change on montane salamanders.

## Graphical abstract

Map of elevation and site locations for study.

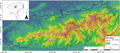

## 1 INTRODUCTION

Climate change is a major force of biotic change worldwide. Rising temperatures and changing patterns of precipitation are known to affect phenology (Blaustein et al., 2001; Todd et al., 2011), species interactions (McKone et al., 1998; Winder & Schindler, 2004), physiology (McCain & Sanders, 2010; Somero, 2010; Wang & Polglase, 1995), diversity (McCain, 2004, 2009; McCain & Grytnes, 2010), and the distribution of species (Laurance et al., 2011; Rowe, 2005; Tingley et al., 2009). Many species exhibit niche tracking, the process of following limiting environmental conditions across time and space (Moritz et al., 2008; Tingley et al., 2009). With warming temperatures, species are expected to move latitudinally toward the poles and higher in elevation in montane regions (Boisvert‐Marsh et al., 2014; Scherrer et al., 2017). However, not all species move in directions predicted by changing temperature due to other factors such as precipitation, land cover change, or competition with resident species (Laurance et al., 2011; Rowe, 2009; Rowe et al., 2010). Understanding the capacity of organisms to adapt and shift geographic ranges in response to rapid climate change is a major challenge facing ecologists and conservation practitioners who want to take action to protect or manage species (Loarie et al., 2008), and these adaptations, or lack thereof, can lead to variation in abundance across elevations.

Montane regions offer advantages for studying changes in animal distributions associated with climate change. Climate change effects are expected to be observed earlier and more consistently over elevational gradients than over larger latitudinal gradients (Sekercioglu et al., 2008; Shoo et al., 2006; Sodhi et al., 2008). Additionally, the steepness of the gradient allows researchers to conduct more thorough surveys compared with having to cover much larger areas across a species’ latitudinal gradient. Finally, montane regions have the potential to compress species at the tops of the mountains and create “sky islands” for species unable to move between suitable mountaintop habitats (Gifford & Kozak, 2012; Laurance et al., 2011; Milanovich et al., 2010; Urban, 2018).

There has been little consensus on which traits impact a species’ ability to shift their range in response to changing climates (Angert et al., 2011). The ability to track an existing climatic niche may be influenced by dispersal capabilities, life history, behavioral traits, diet breadth, specific habitat needs, landscape characteristics, or rate of climate shift (Burrows et al. 2014, Urban, 2015). Laurance et al. (2011) found high elevation specialists are at greater risk of extinction due in part to the higher frequency of specialization among ectotherms compared with endotherms. As such, groups like montane salamanders may be especially vulnerable to climate‐induced extinction. The southern Appalachian Mountains in eastern North America are home to one of the greatest diversities of salamanders in the world (Petranka, 1998). In addition to having high elevation specialists in close proximity to congeneric low elevation species, this region supports species with a variety of life history traits (e.g., from fully aquatic to fully terrestrial) and body sizes (e.g., 0.1–2200 g; Petranka, 1998). The diversity, high endemism, low vagility, and range of life history traits make salamanders in the southern Appalachian Mountains ideal for understanding elevational distributions and responses to climate change. Furthermore, studies have suggested that southern Appalachian salamanders may be declining in abundance and range (Highton, 2005; Adams et al., 2013; Caruso & Lips, 2013; but see Moskwik, 2014) and possibly shrinking in body size (Caruso et al., 2014). However, the baseline for the latter comparison and the accuracy of these estimates have been questioned particularly for the lack of control for observational processes (Connette et al., 2015; Grant, 2015) and the difficulty in assessing whether or not a probabilistic sample selection was used. Biophysical models of *Plethodon* spp. have highlighted the importance that behavior and plasticity may have in shaping salamanders’ ability to cope with shifts in climate patterns (Peterman & Gade, 2017; Riddell et al., 2018, 2019).

Understanding climate effects on species over elevational gradients is hindered by limitations of existing data. There is a wealth of recent and historic presence‐only data from museum records and opportunistic reports, but the lack of systematic surveys limits the utility of these data (Grant, 2015; Kéry et al., 2006). Opportunistic presence‐only data can only be used to determine the minimum extent of a species’ range and resurveying of these areas only provides information about extinctions, not changes in population size. Nor does it provide information on colonization, range expansions, or range shifts because there is no information about where the species did not occur (Tingley & Beissinger, 2009). Additionally, systematic surveys without temporal replication are unable to distinguish nondetections from true absences (MacKenzie et al., 2003) because false absences under‐predict historic range and extinction while over‐predicting colonization (Tingley & Beissinger, 2009), resulting in biased estimates of a species range. Statistical frameworks that use a combination of spatial and temporal replication allow for reduced bias of colonization and extinction estimates (MacKenzie et al., 2002, 2006, 2009) and unbiased estimates of abundance across gradients, even in fluctuating populations (Dail & Madsen, 2011; Royle, 2004; Royle & Dorazio, 2008; Tyre et al., 2003).

Accurate spatial distributions along elevational gradients are critical to understanding the impact of climate change on species ranges and extinction risks, but a lack of adequate abundance data has inhibited the understanding of climate change effects on range shifts and species declines in montane regions (Shoo et al., 2005). Hierarchical models of abundance have great potential for estimating climate‐driven range shifts and predicting the probability of extinction under various climate scenarios. These models offer the possibility of addressing concerns raised by Shoo et al. (2005) while more accurately delineating ranges shifts. Surveys for use with hierarchical *N*‐mixture type models take much less time than mark‐recapture methods allowing for coverage of larger geographical areas (e.g., Royle & Dorazio, 2008; Lyons et al., 2012; Pregler et al., 2019; but see Barker et al., 2018). The sampling can incorporate traditional removal sampling if necessary (e.g., Hocking et al., 2018; Pregler et al., 2019) but does not require it and does not necessarily require handling of specimens, which also makes them suitable for sensitive, difficult to capture, and endangered species.

In this study, we had two primary objectives: (1) estimate salamander abundance along an elevational gradient in Great Smoky Mountains National Park (GSMNP) while accounting for imperfect detection using repeated spatial and temporal surveys; and (2) evaluate environmental and habitat effects on abundance and detection for species with different life history traits. We hypothesize salamander abundance will increase with elevation and that abundance and detection will increase with higher precipitation and humidity.

## 2 MATERIALS AND METHODS

### 2.1 Study site

**Figure 1:**
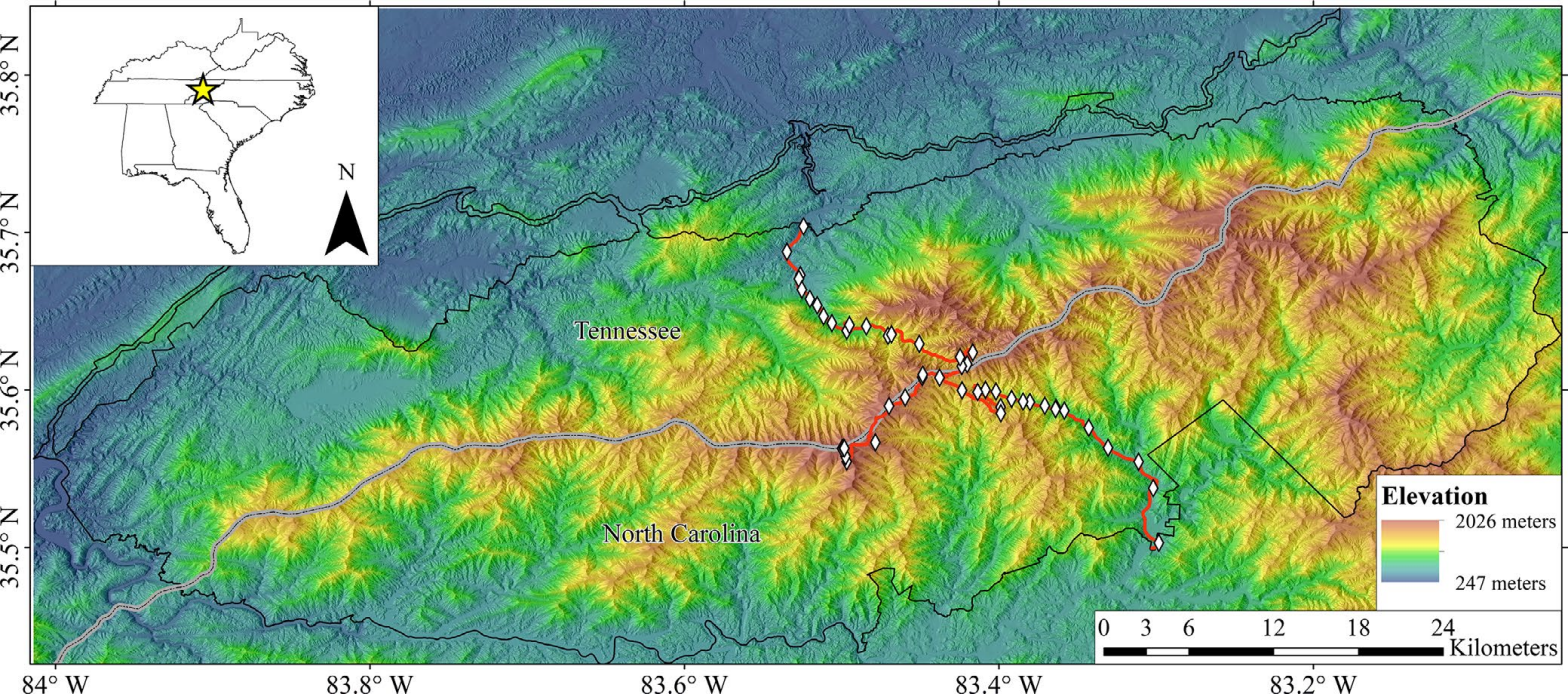
Map depicting the boundary of Great Smoky Mountains National Park in Tennessee and North Carolina, USA. White diamonds indicate the locations of the sample sites distributed across an elevational gradient. All sampling sites were situated in proximity to drivable roadways (red lines)

We identified 70 potential survey sites along an elevational gradient (412–2,025 m a.s.l.) in GSMNP. The GSMNP is located in the Blue Ridge Mountains of the Appalachian Mountain chain in the southeastern United States. The 2,114 km^2^ area ranges in elevation from 260 m to 2025 m, where temperature during the growing season in higher elevation sites ranges from 10 to 15°C (Shanks, 1954). Annual precipitation in GSMNP is among the highest in eastern North America, though seasonal variation in temperature and precipitation is variable leading to lower elevation sites warming significantly greater than higher elevations since 1980 (Lesser & Fridley, 2016). We selected sites along Route 441 from Tennessee to North Carolina and along Spur Road to the top of Clingman's Dome (highest point in GSMNP, third highest peak in eastern USA) that were within 2 km (overland walking distance) of vehicle pull‐offs and a minimum of 800 m from each other to facilitate safety during nighttime surveys. We then randomly selected 45 sites (of the 70 available) for sampling with elevational stratification: 15 at high elevations (1,501–2,025 m a.s.l.), 15 at mid‐elevations (1,001–1,500 m), and 15 at low elevations (412–1,000 m), and before the first survey, we added three additional sites from our initial 70 for a more even distribution over the elevational gradient on both sides of the continental divide. This resulted in 48 sites ranging in elevation from 412 m to 2,025 m (Figure 1). At each site, we established 2–6 transects (mean = 3.3) based on accessibility (total transects = 195). Transects were chosen based on the ability to survey an area at a specific elevation where at least two (but up to 6), 25 × 4 m transects could be created ≥ 50 m from the road and be separated by a minimum of 10 m. Beyond these conditions, transects location and orientation were haphazardly selected. The geographic position (latitude and longitude) of the start and end of each transect was recorded using a handheld GPS (precision ~ 3 m; Garmin 62sc).

At three of the 16 mid‐elevation sites, it was impossible to effectively survey for salamanders due to the density of shrubs (*Rhododendron* spp. and *Leucothoe* spp.). Therefore, we conducted surveys at three mid‐elevation sites on small recreational trails (1–3 m wide). To account for potential differences in detection on trails compared with undisturbed forest habitat, we also conducted adjacent forest and trail transects at select high and low elevation sites (e.g., four forest transects and two trail transects), resulting in 70 of 195 (36%) transects conducted on trails. Prior analysis found no significant differences in species abundance between transects on trails and transects in undisturbed forest habitat (Milanovich et al., 2015).

### 2.2 Salamander sampling

We conducted nighttime visual encounter surveys (VES) along transects to locate surface‐active salamanders—a more effective way to sample lungless salamanders compared to daytime VES (Crawford & Semlitsch, 2007). For each survey, one of four observers (the authors) walked a transect and recorded the number of individuals of each salamander species observed within 2 m of either side of the center transect line, resulting in a 100 m^2^ transect survey area (25 × 4 m). Salamanders were not disturbed except to aid in further identification as needed. For each VES, observers spent 10–20 min to conduct a VES on a transect depending on the terrain, density of vegetation, and number of animals that had to be handled for identification.

We visited between 6 and 16 sites each night (mean = 11.5) over 21 survey nights split between two sampling periods between 13 June and 20 July 2012. Observers were randomly assigned to a transect at each site and then rotated through the transects at each site on future occasions to avoid potential observer bias. A transect was skipped if the surveyor was unavailable to conduct the survey on a given occasion, but our analysis framework accommodates such missing data. On each sampling night we randomly selected 3–5 sites from each of three elevations (stratified random transect selection within elevations; transects were never surveyed more than once on a given sampling occasion). On each night, we randomized the starting site then proceeded in the most convenient route to the other sites. This prevented sampling sites or specific elevations at the same time of night on each occasion. We conducted all surveys between 21:00 and 03:00 hr EDT.

### 2.3 Habitat and environmental measurements

We measured local habitat variables and calculated landscape metrics hypothesized to influence detection and abundance of salamanders (Williams & Berkson, 2004). Along each transect at 5 m intervals, we measured percent canopy cover (during daylight hours) using a spherical densiometer, the proportion of a 1‐m^2^ quadrat covered by vegetation, and leaf litter depth in each corner of each quadrat. We used the mean of these replicate observations to make inferences on abundance and detection at the transect‐level within each site. We derived landscape measures that included calculated slope, distance to nearest stream, topographic position index (TPI), and topographic wetness index (TWI) from 10‐m resolution digital elevation model (DEM) in ArcGIS (v9.3, ESRI). Topographic position represents a transect's slope position relative to the surrounding landscape and was calculated from a 10‐m digital elevation model using a 100‐m moving window (Dilts 2010). Topographic wetness was calculated accounting for solar insolation (azimuth = 180.0, altitude = 75.8; Theobold 2007). At the start of each survey, we recorded air temperature and relative humidity at each site using a handheld weather meter (Kestrel 4,000). We derived spatial rainfall maps describing the 24 hr cumulative precipitation across GSMNP using spatial Kriging of rainfall estimates, based on temporal rainfall measures obtained from 24 weather stations located in GSMNP and the surrounding area.

### 2.4 Analyses

We used *N*‐mixture models to estimate abundance of species within each of our 100‐m^2^ transects while accounting for imperfect detection (Royle, 2004). This model assumes population closure over the duration of the sampling period (5 weeks), but allows abundance to vary in response to transect‐specific covariates and detection probability can vary in time (survey occasion) and space (transect and site). These are long‐lived species (Castanet et al., 1996; Hairston, 1983) and survival over five weeks is close to one. They also are also territorial and have small home ranges (Nishikawa, 1990). Therefore, we are comfortable assuming the super population size does not change over five weeks. The surface activity available to detection varies daily and even hourly in response to daily conditions and additional unknown factors. As a result, we do not distinguish between the observer's visual detection and the individual being on the surface of the leaves or vegetation. The detection is conditional on both of these processes. If multiple observers were used in combination with multiple visits, it would be possible to further partition the surface activity from the observational process, similar to techniques developed by Amundson et al. (2014). In this paper, we have a combined detection process that is a function of environmental and habitat conditions along with additional random variation to account for unmeasured processes.

This model assumes that abundance is distributed following a Poisson distribution and that the probability of detecting an individual is conditional on abundance and distributed following a binomial distribution. Both the abundance and detection parts of the model allow for overdispersion. Model parameters were estimated in a Bayesian framework using Hamiltonian Monte Carlo implemented in Stan via rstan (Stan Development Team, 2020) in the R programming language (R Core Team, 2019). Refer to Appendix S1 for more details regarding implementation.

To incorporate the effects of covariates on abundance and detection, we used log and logit link functions, respectively. The most parameterized model attempted for each species was.

$$N_{i}∼\mathrm{Poisson}\left(\lambda_{i}\right)$$

$$\log_{e}\left(\lambda_{i}\right)=\alpha +\beta_{1}{\mathrm{Elev}}_{i}+\beta_{2}{\mathrm{Elev}}_{i}^{2}+\beta_{3}{\mathrm{TWI}}_{i}+\beta_{4}{\mathrm{Litter}}_{i}+\beta_{5}{\mathrm{logHerb}}_{i}+\beta_{6}{\mathrm{Stream}}_{i}+\varepsilon_{\mathrm{site}}\sigma_{\mathrm{site}}$$

$${\mathrm{Count}}_{\mathrm{ij}}∼\mathrm{Binomial}\left(N_{i},p_{\mathrm{ij}}\right)$$

$$\mathrm{logit}\left(p_{\mathrm{ij}}\right)=\alpha +\beta_{1}{\mathrm{Temp}}_{\mathrm{ij}}+\beta_{2}{\mathrm{Temp}}_{\mathrm{ij}}^{2}+\beta_{3}{\mathrm{Precip}}_{\mathrm{ij}}+\beta_{4}{\mathrm{Humidity}}_{\mathrm{ij}}+\beta_{5}{\mathrm{Herb}}_{i}+{\beta_{6}\mathrm{Herb}}_{i}^{2}+\delta_{\mathrm{ij}}\sigma_{p}$$

where mean abundance at transect *i* (*λ_i_*) is a function of elevation, elevation squared, TWI, leaf litter depth, proportion herbaceous ground cover, and distance to the nearest stream. We also included a random site effect (*ε*_site_) to account for spatial correlation in abundance among transects at a given site. For computational performance in Stan, we used a noncentered parameterization for the random effect where $\varepsilon_{\mathrm{site}}∼N\left(0,1\right)$ is multiplied by the standard deviation $\sigma_{\mathrm{site}}$ (Monnahan et al. 2016). Detection probability was modeled as a logit link function of air temperature, temperature squared, precipitation in the previous 24 hr, relative humidity, proportion herbaceous ground cover, and ground cover squared. We also found that detection was over dispersed with respect to the expectations of a binomial distribution so we included a random overdispersion term (*δ_ij_*) with a standard deviation of $\sigma_{p}$ (Kéry and Schaub 2012), again following a noncentered parameterization. All independent variables used in the model were scaled and centered prior to analysis and had a pairwise Pearson correlation less than 0.60 to avoid problems associated with multicollinearity.

We employed Bayesian methods to estimate the parameters of this model using Stan software in program R (R Core Development Team, 2019) via the rstan package (Stan Development Team. RStan: the R interface to Stan v2.19.3). We used vague normal priors for all abundance coefficients with a mean of zero and standard deviation of 10, and normal priors with a mean of 0 and standard deviation of 2 for detection coefficients to have relatively uniform priors on the true detection scale (Northrup & Gerber, 2018). For the standard deviation of the random effects, we used half‐Cauchy priors with a scale of 2.5. We ran 6 chains, each with 2000 warmup iterations then ran the next 2,000 iterations and saved every other iteration for inference (6,000 total iterations saved). We used the potential scale reduction factor (Vehtari et al., 2020) to test for model convergence as well as visual inspection of the chains. To evaluate model fit, we used posterior predicative checks the observed data compared with idealized (i.e. model‐generated data) data. The research compendium (Marwick et al., 2018) including all data and code used in the paper can be found at https://github.com/djhocking/GSMNP‐Elevation.

## 3 RESULTS

We captured a total of 9,522 salamanders of 14 species (Table 1) across all transects between June and July 2012. Our results show many species had large elevational ranges exceeding 1,000 m. Blue Ridge two‐lined salamander (*Eurycea wilderae*) was the most widely distributed, ranging from our lowest survey location (447 m) to the highest peak in GSMNP (2,025 m; Table 1). It is likely the species occurs at even lower elevations outside the park, beyond our survey transects. Jordan's Salamander (*Plethodon jordani*), Pygmy Salamander *(Desmognathus wrighti),* and Spring Salamander (*Gyrinophilus porphyriticus*) were also observed at the top of Clingman's Dome and had observed ranges in excess of 900 m. There was uncertainty in the field identification and differentiation of Ocoee Salamanders (*Desmognathus ocoee*) and Imitator Salamanders (*D. imitator*) at the beginning of the study. Additionally, the taxonomy of *D. ocoee* is uncertain and may consist of multiple species (Pyron et al. 2020). Finally, *D. ocoee* and *D. imitator* have different reported elevational distributions in GSMNP (Dodd, 2004; Table 1); therefore, we grouped *D. ocoee* and *D. imitator* in our summary tables but did not conduct formal analyses on their independent or combined distributions. Our surveys expanded the known range of *D. wrighti* and *P. jordani* downslope by 88 and 101 m, respectively and extended the known range of Santeetlah Dusky Salamander (*Desmognathus santeetlah*; occurs at 1,790 m) and *E. wilderae* upslope by 99 and 242 m, respectively, within GSMNP (Table 1).

**Table 1:** Number of captures and minimum and maximum elevations (meters above sea level) from this study in GSMNP during June–July 2012, along with minimum and maximum elevations reported by Dodd (2004) sampled between 1998 and 2001 and records in the Global Biodiversity Information Facility (GBIF) and VertNet databases. All records are restricted to GSMNP, and species may have different ranges outside the park

Species	No. captured	Observed in this study		Observed in Dodd, 2004		GBIF & VertNet	
		Min	Max	Min	Max	Min	Max
*Desmognathus conanti*	2	678	678	~340	960	503^c^	2003^c^
*D. imitator/ocoee*	1,059	678	2022	756/866	1800/1830	521/534	2003/1819
*D. monticola*	23	678	1566	381	1646	391	1,171
*D. quadramaculatus*	3	1,499	1717	341	1714 (1829)^b^	481	1582
*D. santeetlah*	13	678	1893	402	1,790	521	1788
*D. wrighti*	858	671	2022	762	2025	843	2025
*Eurycea wilderae*	1,021	447	2020	335	1783	344	1,840
*Gyrinophilus porphyriticus*	12	1,057	2021	~300	2025	396	1819
*Notophthalmus viridescens*	1	666	666	~300	663 (975)^b^	NA^d^	NA^d^
*Plethodon glutinosus* ^a^	4	670	1,168	585	1,280	397	1,375
*P. jordani*	6,399	678	2022	775	2025	810	2025
*P. serratus*	2	654	843	360	1527	447	1646
*P. teyahalee* ^a^	125	671	1,382	649	1516	515	1713

^a^ *Plethodon glutinosus* and *P. teyahalee* may only be confidently distinguished by molecular means in some areas with GSMNP.

^b^ Higher elevations reported in Dodd (2004 not found during current surveys noted parenthetically.

^c^ Included all records of *Desmognatus fuscus* from the GBIF and HerpNet databases because *D. conanti* was not recognized as a distinct species at the time most records were collected.

^d^ Excluded because fewer than 10 records within GSMNP with sufficient information on location or elevation.

**Figure 2:**
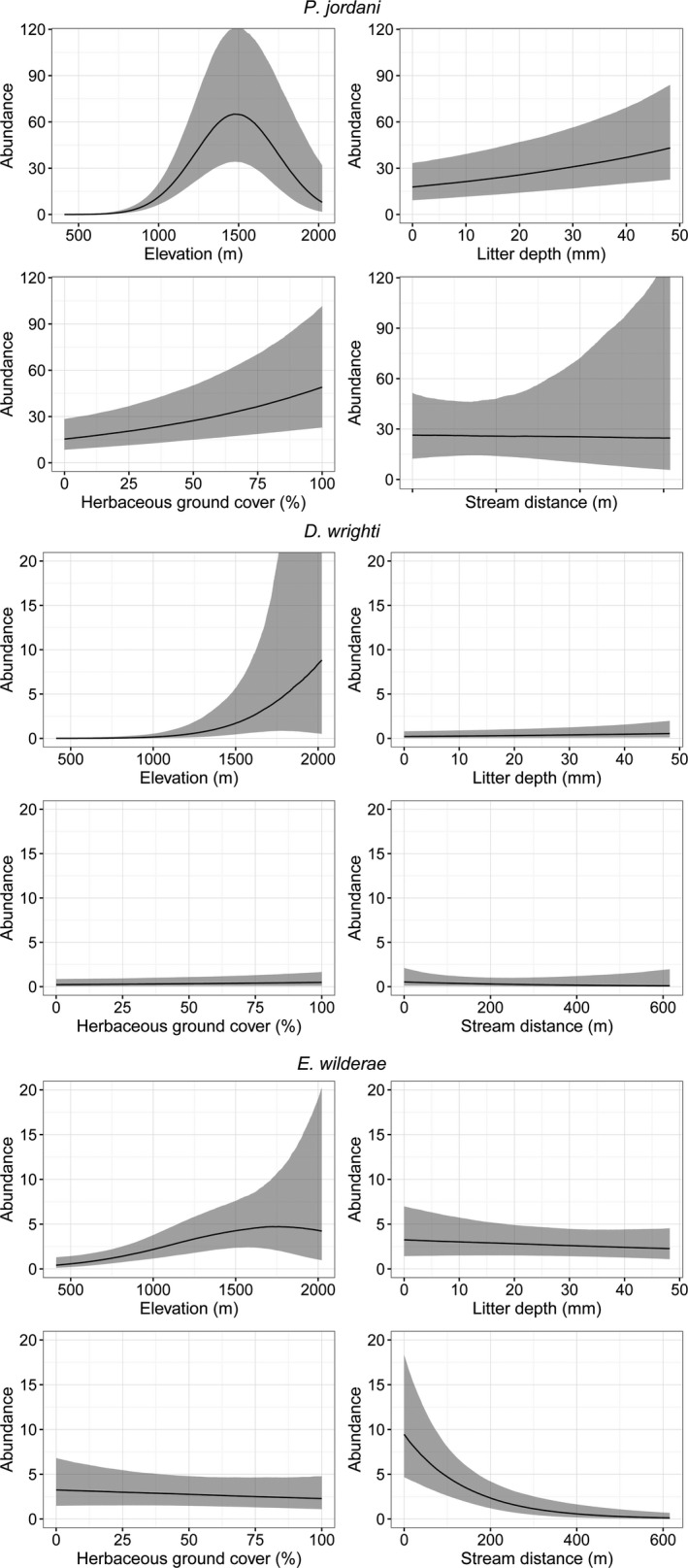
The conditional posterior probabilities for the effects of elevation, litter depth, herbaceous ground cover, and stream distance on *Plethodon jordani*, *Desmognathus wright*, and *Eurycea wilderae* abundance. The black line represents the median estimate, and the gray area represents a 90% credible interval (5%–95% posterior probability)

Of the 14 species captured, *P. jordani*, *D. wrighti*, and *E. wilderae* were observed in sufficient numbers to model abundance across sites accounting for imperfect detection. Based on posterior predictive checks (Appendix S2), the models for all three species adequately fit the data. None of our models exhibited any pathological behavior or divergencies, and no iterations saturated the maximum tree depth of 10. Based on all 24,000 postwarmup iterations, the maximum $\hat{R}$ value was 1.004, the minimum bulk effective sample size was 1,675, and the minimum tail effective sample size was 2,564 across all parameters for *P. jordani* (Vehtari et al., 2020). The posterior predictive checks indicated good model fit based on the relationship between expected counts from the model and observed counts (Appendix S2). All parameter estimates are found in Table 2. Regarding parameters with 90% CRI not overlapping zero, the abundance of *P. jordani* had a large quadratic relationship with elevation in the model (Table 2). Ground cover and litter depth positively influenced *P. jordani* abundance, *D. wrighti,* and *E. wilderae* tended to be more abundant at higher elevations, and *E. wilderae* abundance decreased exponentially with distance from the nearest stream (Figure 2).

**Table 2:** Coefficient estimates from *N*‐mixture model of abundance accounting for imperfect detection for the three most frequently observed species. All independent variable data were standardized by subtracting the mean and dividing by the standard deviation, making coefficient estimates comparable within and among species

Variable	*Plethodon jordani*			*Desmognathus wrighti*			*Eurycea wilderae*		
	Mean	2.5%	97.5%	Mean	2.5%	97.5%	Mean	2.5%	97.5%
Abundance									
N‐intercept	3.254	2.524	3.971	−1.193	−2.978	0.240	1.014	0.287	1.680
Elevation	2.467	1.736	3.252	2.503	0.919	4.521	0.796	0.151	1.463
Elevation^2^	−1.658	−2.321	−1.071	−0.375	−1.716	0.851	−0.298	−0.831	0.250
TWI	0.016	−0.101	0.135	0.136	−0.083	0.356	0.063	−0.138	0.274
Litter Depth	0.215	0.071	0.360	0.223	−0.092	0.538	−0.084	−0.363	0.200
Ground Cover	0.322	0.121	0.519	0.207	−0.117	0.565	−0.095	−0.404	0.233
Stream Dist	−0.004	−0.536	0.599	−0.464	−1.680	0.691	−1.116	−1.769	−0.489
Site *SD* (σ_site_)	1.464	1.064	2.008	2.839	1.916	4.194	1.268	0.921	1.729
Detection probability									
p‐intercept	−1.020	−1.453	−0.534	−1.970	−2.546	−1.402	−1.660	−2.195	−1.150
Temperature	0.216	0.031	0.393	−0.120	−0.844	0.588	0.125	−0.293	0.533
Temperature^2^	−0.130	−0.251	−0.016	−0.285	−0.711	0.124	−0.478	−0.804	−0.177
24‐hr Precip	0.057	−0.027	0.142	0.420	0.142	0.715	0.413	0.184	0.660
Ground Cover	−0.520	−0.751	−0.271	0.536	0.034	1.043	0.385	−0.074	0.843
Ground Cover^2^	0.172	0.047	0.297	−0.210	−0.542	0.119	−0.202	−0.494	0.095
Rel. Humidity	0.109	−0.004	0.220	0.930	0.476	1.440	0.684	0.346	1.050
Random Obs. *SD* (σ_site_)	0.759	0.636	0.927	1.818	1.412	2.306	1.761	1.379	2.204

**Figure 3:**
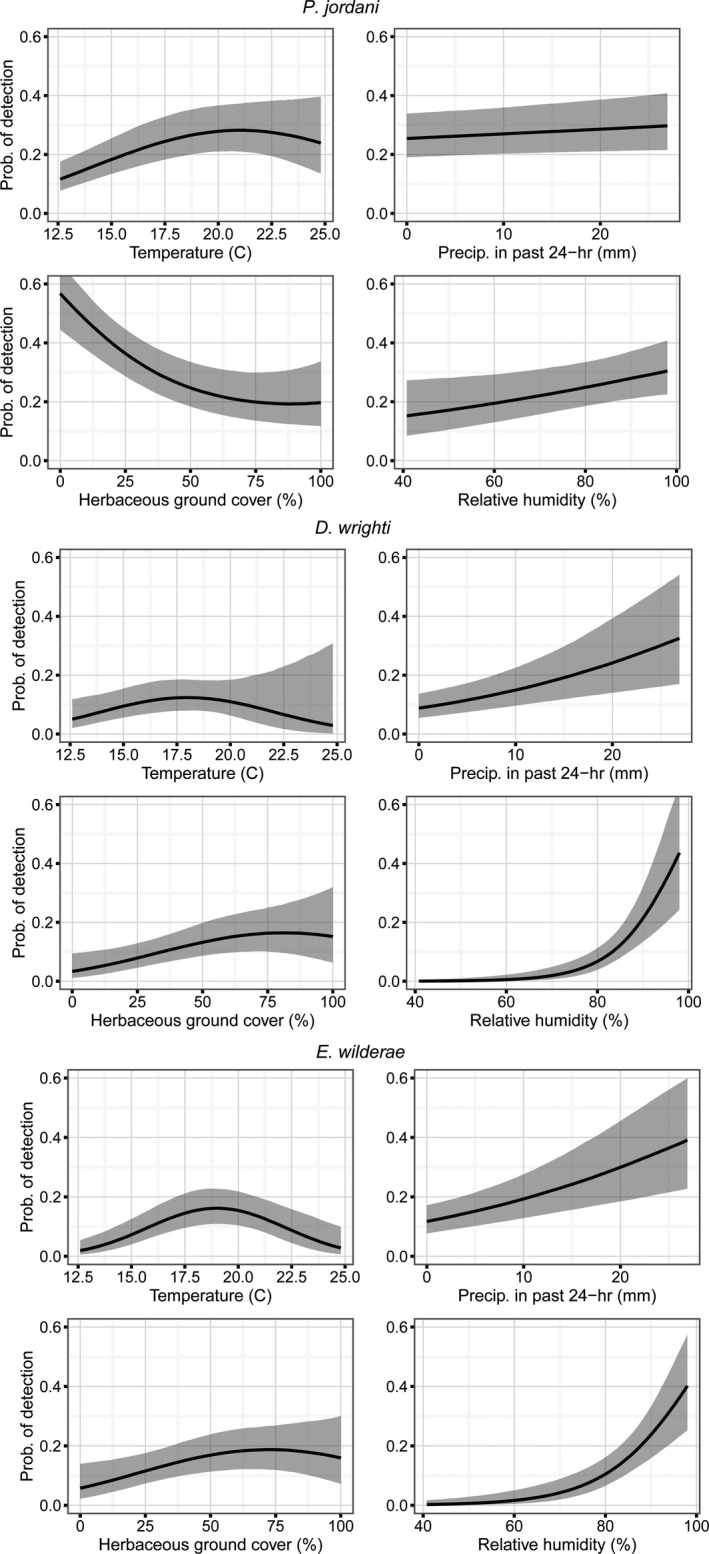
The conditional posterior probabilities for the effects of elevation, litter depth, herbaceous ground cover, and stream distance on *Plethodon jordani*, *Desmognathus wright*, and *Eurycea wilderae* detection. The black line represents the median estimate, and the gray area represents a 90% credible interval (5%–95% posterior probability)

We also jointly estimated the effects of environmental conditions on the probability of detecting an individual. The mean detection rate across visits and sites was 0.26, 0.12, and 0.17 for *P. jordani*, *D. wright*, and *E. wilderae,* respectively. Relative humidity generally had positive effects on the detection of individuals of all three species, but was most influential for the smaller *D. wrighti* and *E. wilderae* (Table 2). *Plethodon jordani* detection was affected by temperature and ground cover with detection being highest when ground cover was 0% and temperature was 20°C (Figure 3). *Eurycea wilderae* was optimally detected at 19°C (Figure 3). Both *D. wrighti* and *E. wilderae* detection increased with increasing amount of precipitation in the previous 24 hr and *D. wrighti* detection increased with increasing proportions of herbaceous ground cover (Table 2, Figure 3).

## 4 DISCUSSION

We used repeated spatial and temporal surveys to account for imperfect detection and generate estimates of abundance of salamanders over an elevational gradient in Great Smoky Mountains National Park. Elevation was an important predictor of abundance for all three species with sufficient data for analysis. *Plethodon jordani* exhibited a mid‐elevation peak, *D. wrighti* abundance increased exponentially with increasing elevation, while *E. wilderae* increased with elevation up to approximately 1,600 m before abundance estimates leveled off. However, there is considerable uncertainty in the effects of higher elevations in all species (1500–2025 m; Figures 2 and 3). This may be due to a decrease of stream habitat at higher elevations (needed by *E. wilderae* for reproduction) or insufficient data to precisely model abundance at higher elevations. The abundance of terrestrial *Plethodon* spp. is closely tied to streams at low elevations but is independent of streams at high elevations where cool, moist climates are more continuously distributed across the landscape (Gade et al., 2020; Gade & Peterman, 2019).

Climate niche conservatism over evolutionary time has driven the mid‐elevation peak in amphibian species richness and makes amphibians particularly vulnerable to rapid climate change (Farallo et al., 2020; Kozak & Weins, 2010). In GSMNP, Gifford and Kozak (2012) found that physiological constraints limit the lower elevation of *P. jordani* and it is possible that a similar mechanism operates with *D. wrighti*. It is unclear what processes underlie the mid‐elevation peak in abundance for *P. jordani*. This pattern could be driven by physiological constraints related to temperature and precipitation (McCain & Sanders, 2010), but there has been little evidence for the metabolic theory of ecology shaping herpetofauna distributions along elevational gradients (McCain & Sanders, 2010). It is also possible that limited area at higher elevations makes it less likely for species to occur at high elevations due to limited habitat space and isolation shifting colonization‐extinction dynamics and genetic diversity (McCain, 2003; McCain & Grytnes, 2010; Rowe, 2009). This space limitation could also increase the intensity of interspecific interactions (Hairston, 1986; McCain & Grytnes, 2010). For the Southern Appalachian Salamander (*Plethodon teyahalee*), competition with *P. jordani* has been indicated as the upper elevation limitation (Gifford & Kozak, 2012), and it is possible that competitive interactions limit the upslope distribution of *E. wilderae*. Habitat limitations present a final hypothesis as to the processes leading to this mid‐elevation peak (McCain & Grytnes, 2010; Rowe, 2007). For a species with a complex life cycle (Wilbur, 1980), it is also possible that the tops of mountains lack suitable stream breeding habitat, so they are less likely to migrate greater than 300 m from streams (Crawford & Semlitsch, 2007). The other two species modeled are fully terrestrial and therefore are not limited by distances between complementary habitats (Dunning et al., 1992; Hocking & Semlitsch, 2007; Pope et al., 2000), although microclimate dependencies are likely to shape the distribution of abundance across the landscape (Gade & Peterman, 2019). Overall, it is unlikely that a single driver controls the elevational distribution of any of these species, even for a taxon so physiologically linked to temperature and moisture, but rather some combination of processes leads to the observed distributions (McCain & Grytnes, 2010; Rowe, 2009; Tingley et al., 2012) ) and warrants future mechanistic, manipulative studies.

Numerous species have altered their elevational ranges in response to climate change and other environmental factors. Birds have been found to track their climatic niche over the past century (Tingley et al., 2009). Small mammals have experienced climate‐related elevational range changes. Low elevation small mammals have expanded their range while high elevation species have contracted their ranges in Yosemite National Park over the past century (Moritz et al., 2008). However, other small mammal distributions have been affected as much by changes in land‐use as by climatic changes in the 20th century (Rowe, 2007; Rowe et al., 2010). Among amphibians, there have been major climate‐induced declines, particularly among high elevation species (Pounds & Crump, 1994; Rovito et al., 2009). In the southern Appalachian Mountains, increases in temperature (11.8 – 14.2°C) from 1974 to 1990 caused an upslope shift in the hybrid zone between Red‐legged Salamander (*Plethodon shermani*) and *P. teyahalee*, resulting in fewer pure individuals of the high elevation specialist, *P. shermani* (Walls, 2009). Additionally, temperature is not the only climate driver that influences elevational ranges; precipitation can have large effects on distributions (Gillings et al., 2015; Reich et al., 2014; Rockwell et al., 2017). This is particularly true for amphibians, which require environmental moisture for respiration and to prevent desiccation. Many species also have complex life cycles that require water with appropriate flow or hydroperiod for reproduction, both of which are affected by temperature and precipitation. High correlations between temperature and moisture observed in this study prevented their separation in the models and elevation served as a proxy for their combined effects. Future changes in elevational ranges and abundances will likely be a result of interactions between changes in temperature, precipitation, and land cover (particularly forests through shading and evapotranspiration), as they could influence the environmental temperature and moisture experienced by amphibians in addition to affecting stream flow and wetland hydroperiod.

Habitat conditions affect amphibian distributions and abundances. In addition to elevation, *P. jordani* were also found in greater abundance on sites with more ground cover and deeper leaf litter. This is similar to previous research, which found *P. shermani* (Gade & Peterman, 2019), Seal Salamander (*Desmognathus monticola*), *D. ocoee*, and *E. wilderae* were all positively associated with leaf litter (Crawford & Semlitsch, 2008). Deeper leaf litter likely provides a variety of benefits for *Plethodon* and *Desmognathus* spp. including increased invertebrate abundance as a food source (Coleman et al., 2004; Petranka, 1998) and cool, moist microhabitats that prevent desiccation (Crawford & Semlitsch, 2008; Peterman & Semlitsch, 2013, 2014; Rittenhouse et al., 2008). We did not identify other variables that influenced *D. wrighti* abundance and it had the largest random variation among sites, indicating that there are other environmental and habitat conditions controlling their local distributions. They are typically associated with cove forests and the specific topography, soils, forest cover, and aspect could all interact in complex ways to influence their local abundance (Hairston, 1949; Organ, 1961; Tilley & Harrison, 1969), but that also may be related to elevation. *Eurycea wilderae* occurred in higher abundance at sites close to streams, which was expected based on breeding habitat requirements and previous research showing that the majority of a population is typically within 43 m of a stream (Crawford & Semlitsch, 2007).

By incorporating site‐specific variables related to abundance, our abundance estimates for species across the elevational gradient resulted in smoother, continuous abundance‐elevation relationships (Figure 2). Improving abundance estimates and reducing bias by accounting for imperfect detection is critical in evaluating population declines and range shifts. Without accounting for the imperfect detection, the observation and ecological processes are confounded, thereby obfuscating changes in population state. This confounding of uncertainties (Nichols et al., 2011) can reduce the ability to detect population trends or changes in range edges or centroids (Tingley & Beissinger, 2009), or obscure contact zones where species interact (Amburgey et al., 2019). This is especially important in monitoring programs and assessments of at risk species, such as high elevation species, which have been suggested as especially vulnerable to climate change (Amburgey et al., 2019; Gifford & Kozak, 2012; Laurance et al., 2011; Sekercioglu et al., 2008; Sodhi et al., 2008); ectothermic vertebrates, such as amphibians, have a disproportionally high number of high elevation specialists compared with other taxa (Laurance et al., 2011). Amphibians, particularly salamanders, are also difficult to observe, owing to the small size, cryptic coloration, and fossorial nature of many species. Their activity is also a function of environmental conditions (Keen, 1984; O’Donnell et al., 2015). Therefore, accounting for variability in the activity and observation process is critical in understanding the true abundance and distributions of amphibian species. For these reasons, there has been considerable concern recently regarding the utility of analyses not accounting for imperfect detection when making inference about species abundances and distributions (e.g., Grant, 2015; O’Donnell et al., 2015; Royle & Dorazio, 2008). The use of historical presence‐only or single‐visit presence‐absence data limits the ability to make inference about range changes over time (Grant, 2015; Tingley et al., 2009). We lacked systematic historical data to evaluate range changes over time, but we have now established a rigorous method of sampling and analysis to detect future changes in abundance and distribution in this ecologically significant region.

In addition to improving the precision of abundance estimates and reducing bias, important information can be gained from modeling the detection process—including modeling detectability (Graham & Weinstein, 2018). This is particularly true when detection is more a function of animal activity and less influenced by observer traits (e.g., ability to see or hear species), or at least when these components of detection can be separated. In our study, temperature, precipitation, relative humidity, and herbaceous ground cover all influenced the probability of detection (Table 2). All of these variables, with the exception of ground cover, are likely more related to whether or not salamanders become active on the surface and have little influence on the observer's ability to locate individuals. Therefore, we can infer that *P. jordani* and *E. wilderae* exhibited an optimal temperature for surface activity at approximately 19–20°C), as indicated by the significant negative squared term in the detection submodel. This has similarly been identified for the Red‐backed Salamander (*Plethodon cinereus*) in the northeastern United States (Hocking et al., 2013). We did not find a significant effect of temperature on the detection of *D. wrighti* over the range of observed temperatures; however, it is possible that over a larger range of temperatures or with more data we would identify an optimal temperature for activity. This seems likely as the mean estimated coefficients followed similar patterns to those of the other species but with reduced and more equivocal effects (Table 2). Additionally, there are likely interactions with precipitation and temperature (Spotila, 1972), which we could not assess with our current data. We did find large effects of both precipitation and relative humidity on detection of *D. wrighti*, as well as on *E. wilderae*. The amount of moisture in the air is a critical determinant of salamander activity generally, dictating the rate at which water is lost through evaporation (Peterman & Gade, 2017; Riddell et al., 2018). Relative humidity was also an important predictor of *P. jordani* detection, but the effect size was not as large as with the other two species (Table 2).

Our data also support the use of nighttime VES for estimating lungless salamander population sizes and to examine long‐term trends in populations. For example, our total capture numbers within a five‐week period nearly matched numbers from a much greater effort across five years conducted using daytime surveys (Dodd & Dorazio, 2004). Furthermore, our captures were measurably higher compared with other short‐term studies in the southern Appalachians that relied on cover object searches rather than VES (Bailey et al., 2004; Caruso & Lips, 2013). When considering long‐term plans for analyses of the impacts of global change on species, it is important to match the sampling methods to the natural history of the species of interest. If the detection probability is too low, even hierarchical abundance models accounting for detection cannot calculate accurate abundances (Dail & Madsen, 2011). In the case of southern Appalachian plethodontid salamanders, conducting VES on humid nights for these nocturnal species maximizes detection probability leading to more precise abundance estimates over time. Without accounting for variations in detection, any changes in observed counts through time are interpreted as changes in the population abundance and lead to incorrect inference regarding elevational shifts and conservation options. These sampling methodologies are particularly important to consider when quantifying the predicted impact of global climate change, as mechanistic models utilizing physiological metrics to predict suitable climatic habitat suggest montane salamanders may have a greater proportion of predicted habitat compared with correlative‐based models (Lyons & Kozak, 2020). Thus, providing accurate measures of abundance and detection can help determine what predictions are more valid.

The diversity of lungless salamanders in the Appalachian Highlands is vast, and distributions and surface activity vary across species as a function of habitat structure, temperature, and precipitation. To understand the realized or potential consequences of global climate change, a rigorous and defensible abundance baseline must be established. Our study of GSMNP plethodontid salamanders sets such a baseline (Table 1). While continued monitoring is necessary to track changes in abundance, more in‐depth research, such as capture‐mark‐recapture, is also required to understand the potential mechanisms underlying observed changes (e.g., Caruso & Rissler, 2019). Global climate change is progressing rapidly, and montane plethodontid salamander populations may already be changing. Only rigorous population monitoring can bring such findings to light.

## CONFLICT OF INTERESTS

The authors do not have any competing interest.

## AUTHOR CONTRIBUTIONS

**Daniel Hocking:** Conceptualization (equal); Data curation (equal); Formal analysis (equal); Funding acquisition (equal); Investigation (equal); Methodology (equal); Project administration (equal); Resources (equal); Software (equal); Supervision (equal); Validation (equal); Visualization (equal); Writing‐original draft (equal); Writing‐review & editing (equal). **John Crawford:** Conceptualization (equal); Data curation (equal); Formal analysis (equal); Funding acquisition (equal); Investigation (equal); Methodology (equal); Project administration (equal); Resources (equal); Software (equal); Supervision (equal); Validation (equal); Visualization (equal); Writing‐original draft (equal); Writing‐review & editing (equal). **William E. Peterman:** Conceptualization (equal); Data curation (equal); Formal analysis (equal); Funding acquisition (equal); Investigation (equal); Methodology (equal); Project administration (equal); Resources (equal); Software (equal); Supervision (equal); Validation (equal); Visualization (equal); Writing‐original draft (equal); Writing‐review & editing (equal). **Joseph Milanovich:** Conceptualization (equal); Data curation (equal); Formal analysis (equal); Funding acquisition (equal); Investigation (equal); Methodology (equal); Project administration (equal); Resources (equal); Software (equal); Supervision (equal); Validation (equal); Visualization (equal); Writing‐original draft (equal); Writing‐review & editing (equal).

## Supporting information

Appendix S1

Appendix S2

## Data Availability

Data are available via Dryad at https://doi.org/10.5061/dryad.280gb5mp2.
