# Supplementary material for: Abundance of montane salamanders over an elevational gradient
Source: Ecol Evol. 2020 Dec 29;11(3):1378–91. doi: 10.1002/ece3.7142 (PMC7863398; doi:10.1002/ece3.7142)
Supplement: Supplementary file 1 — Appendix S1 [file ECE3-11-1378-s001.pdf]

# Appendix A: Model Code

## Stan Model Code

```
// Binomial mixture model with covariates
data {
  int<lower=0> R;       // Number of transects
  int<lower=0> T;       // Number of temporal replications
  int<lower=0> nsites;       // Number of sites
  int<lower=1> sites[R];       // vector of sites
  int<lower=0> y[R, T]; // Counts
  vector[R] elev;          // Covariate
  vector[R] elev2;          // Covariate
  vector[R] litter;          // Covariate
  vector[R] twi;          // Covariate
  vector[R] stream;          // Covariate
  matrix[R, T] RH;      // Covariate
  matrix[R, T] precip;      // Covariate
  matrix[R, T] temp;      // Covariate
  matrix[R, T] temp2;      // Covariate
  vector[R] gcover;      // Covariate
  vector[R] gcover2;      // Covariate
  int<lower=0> K[R];       // Upper bound of population size
}

transformed data {
  int<lower=0> max_y[R];
  int<lower=0> N_ll;
  int tmp[R];

  for (i in 1:R) {
    max_y[i] = max(y[i]);
    tmp[i] = K[i] - max_y[i] + 1;
  }
  N_ll = sum(tmp);
}

parameters {
  real alpha0;
  real alpha1;
  real alpha2;
  real alpha3;
  real alpha4;
  real alpha5;
  real alpha6;
  real beta0;
  real beta1;
  real beta2;
  real beta3;
```

```
  real beta4;
  real beta5;
  real beta6;

  vector[nsites] eps;            // Random site effects
  real<lower=0> sd_eps;
  matrix[R, T] delta;            // Random transect-visit effects
  real<lower=0> sd_p;
}

transformed parameters {
  vector[R] log_lambda; // Log population size
  matrix[R, T] logit_p; // Logit detection probability

  for (i in 1:R) {
    log_lambda[i] = alpha0 + alpha1 * elev[i] + alpha2 * elev2[i] + alpha3 * twi[i] + alpha4 * litter[i]
    for (t in 1:T) {
      logit_p[i,t] = beta0 + beta1 * temp[i,t] + beta2 * temp2[i,t] + beta3 * precip[i,t] + beta4 * gcov
    }
  }
}

model {
  // Priors
  alpha0 ~ normal(0, 10);
  alpha1 ~ normal(0, 10);
  alpha2 ~ normal(0, 10);
  alpha3 ~ normal(0, 10);
  alpha4 ~ normal(0, 10);
  alpha5 ~ normal(0, 10);
  alpha6 ~ normal(0, 10);

  beta0 ~ normal(0, 2);
  beta1 ~ normal(0, 2);
  beta2 ~ normal(0, 2);
  beta3 ~ normal(0, 2);
  beta4 ~ normal(0, 2);
  beta5 ~ normal(0, 2);
  beta6 ~ normal(0, 2);

  eps ~ normal(0, 1);
  sd_eps ~ cauchy(0, 2.5);

  for (i in 1:R) {
    for (t in 1:T) {
      delta[i,t] ~ normal(0, 1);
    }
  }

  sd_p ~ cauchy(0, 2.5); // even this might be more heavily tailed than we want on p. maybe half normal

  // Likelihood
  for (i in 1:R) {
    vector[K[i] - max_y[i] + 1] lp;
```

```
    // vectorized over T
    for (j in 1:(K[i] - max_y[i] + 1))
    lp[j] = poisson_log_lpmf(max_y[i] + j - 1 | log_lambda[i])
    + binomial_logit_lpmf(y[i] | max_y[i] + j - 1, logit_p[i]);
    target += log_sum_exp(lp);
  }
}

generated quantities {
  int N[R] = max_y;   // Abundance (must be at least max_y)
  int N_total;
  vector[R] log_lik;
  real mean_abundance;
  real mean_detection;
  real mean_p;
  vector[R] mean_p_site;
  real fit = 0;
  real fit_new = 0;
  matrix[R, T] p;
  int counter[R];

  for (i in 1:R) {
    // calculate vector logliklihood for use in loo for model comparison
    vector[K[i] - max_y[i] + 1] ll;

    for (k in 1:(K[i] - max_y[i] + 1)) {
      ll[k] = poisson_log_lpmf(max_y[i] + k - 1 | log_lambda[i]) // remake lp because can't use from mo
      + binomial_logit_lpmf(y[i] | max_y[i] + k - 1, logit_p[i]);
    }
    log_lik[i] = log_sum_exp(ll); // for use in loo and multimodel comparison

    // Calculate Abundance - Restrict N to be at least as big as the number of animals observed on a si
      N[i] = poisson_log_rng(log_lambda[i]);
      counter[i] = 0;
      while (N[i] < max_y[i]) {
        N[i] = poisson_log_rng(log_lambda[i]);
        counter[i] += 1;
        if (counter[i] > 100) break;
      }

    p[i, 1:T] = inv_logit(logit_p[i, 1:T]);
  }

    for (i in 1:R) {
    mean_p_site[i] = mean(p[i]);

    for (j in 1:T) {
      // Simulate new data from model for posterior predictive checks
      y_new[i, j] = binomial_rng(N[i], p[i, j]);
    }
    y_new_sum[i] = sum(y_new[i]);
  }
```

```
  N_total = sum(N);  // Total pop. size across all sites
  mean_abundance = exp(alpha0);
  mean_detection = 1 / (1 + exp(-1 * beta0));
  mean_p = mean(p);
}
```

## R Code

Implementation of Stan $N$-mixture model through R.

```
## Random site effects on abundance and random binomial overdispersion with random transect-visit effec

# Based on code by Hiroki ITÔ translated from chapter 12 of Kery and Schuab Bayesian Population Analysi

# non-center as described by Monnahan et al. 2017 MEE

# Load Libraries
library(rstan)
library(dplyr)

# Settings
rstan_options(auto_write = TRUE)
options(mc.cores = parallel::detectCores())
# set.seed(123)

load(file = "Data/Derived/settings.RData")

## Read data
load("Data/Derived/stan_prep.RData")


## Parameters monitored
params <- c("N_total",
            "alpha0",
            "alpha1",
            "alpha2",
            "alpha3",
            "alpha4",
            "alpha5",
            "alpha6",
            "beta0",
            "beta1",
            "beta2",
            "beta3",
            "beta4",
            "beta5",
            "beta6",
            "sd_eps",
            "sd_p",
            "N",
            "mean_abundance",
            "mean_detection",
            "mean_p",
            "log_lik",
            "y_new",
```

```
            "y_new_sum")

## Initial values
inits <- lapply(1:nc, function(i)
  list(alpha0 = runif(1, -1, 1),
       alpha1 = runif(1, -1, 1),
       alpha2 = runif(1, -1, 1),
       alpha3 = runif(1, -1, 1),
       alpha4 = runif(1, -1, 1),
       alpha5 = runif(1, -1, 1),
       beta0 = runif(1, -1, 1),
       beta1 = runif(1, -1, 1),
       beta2 = runif(1, -1, 1),
       beta3 = runif(1, -1, 1),
       beta4 = runif(1, -1, 1),
       beta5 = runif(1, -1, 1),
       sd_eps = runif(1, 0, 1)))

## Call Stan from R

#----- EWIL -----
if(!dir.exists("Results/Stan")) dir.create("Results/Stan", recursive = TRUE)
site_od_full_ewil <- stan("Code/Stan_Models/final_od.stan",
                          data = list(y = EWIL5,
                                      R = nrow(EWIL5),
                                      T = ncol(EWIL5),
                                      nsites = n.sites,
                                      sites = Data5$site_stan,
                                      elev = elev5,
                                      elev2 = elev5^2,
                                      litter = litter5,
                                      twi = twi5,
                                      precip = precip5,
                                      stream = stream5,
                                      # stream2 = stream5 * stream5,
                                      gcover = gcover5,
                                      gcover2 = gcover5^2,
                                      RH = RH5,
                                      temp = temp5,
                                      temp2 = temp5^2,
                                      K = K_ewil),
                          init = inits,
                          pars = params,
                          chains = nc, iter = ni, warmup = nb, thin = nt,
                          # seed = 1,
                          open_progress = FALSE,
                          verbose = TRUE)

if(!dir.exists("Results/Stan")) dir.create("Results/Stan", recursive = TRUE)
saveRDS(site_od_full_ewil, file = "Results/Stan/final_od_ewil_hmc.Rds")


#----- DWRI -----
```

```
site_od_full_dwri <- stan("Code/Stan_Models/final_od.stan",
                          data = list(y = DWRI5,
                                      R = nrow(DWRI5),
                                      T = ncol(DWRI5),
                                      nsites = n.sites,
                                      sites = Data5$site_stan,
                                      elev = elev5,
                                      elev2 = elev5^2,
                                      litter = litter5,
                                      twi = twi5,
                                      precip = precip5,
                                      stream = stream5,
                                      # stream2 = stream5 * stream5,
                                      gcover = gcover5,
                                      gcover2 = gcover5^2,
                                      RH = RH5,
                                      temp = temp5,
                                      temp2 = temp5^2,
                                      K = K_dwri),
                          init = inits,
                          pars = params,
                          chains = nc, iter = ni, warmup = nb, thin = nt,
                          # seed = 1,
                          open_progress = FALSE,
                          verbose = TRUE)

saveRDS(site_od_full_dwri, file = "Results/Stan/final_od_dwri_hmc.Rds")


#----- PJOR -----

site_od_full_pjor <- stan("Code/Stan_Models/final_od.stan",
                          data = list(y = PJOR5,
                                      R = nrow(PJOR5),
                                      T = ncol(PJOR5),
                                      nsites = n.sites,
                                      sites = Data5$site_stan,
                                      elev = elev5,
                                      elev2 = elev5^2,
                                      litter = litter5,
                                      twi = twi5,
                                      precip = precip5,
                                      stream = stream5,
                                      # stream2 = stream5 * stream5,
                                      gcover = gcover5,
                                      gcover2 = gcover5^2,
                                      RH = RH5,
                                      temp = temp5,
                                      temp2 = temp5^2,
                                      K = K_pjor),
                          init = inits,
                          pars = params,
                          chains = nc, iter = ni, warmup = nb, thin = nt,
                          # seed = 1,
```

```
                          open_progress = FALSE,
                          verbose = TRUE)
saveRDS(site_od_full_pjor, file = "Results/Stan/final_od_pjor_hmc.Rds")


#----- Cleanup -----

rm(list = ls())
```
