# Supplementary material for: Abundance of montane salamanders over an elevational gradient
Source: Ecol Evol. 2020 Dec 29;11(3):1378–91. doi: 10.1002/ece3.7142 (PMC7863398; doi:10.1002/ece3.7142)
Supplement: Supplementary file 2 — Appendix S2 [file ECE3-11-1378-s002.pdf]

Appendix B: HMC Diagnostics

Plethodon jordani

View Traceplots

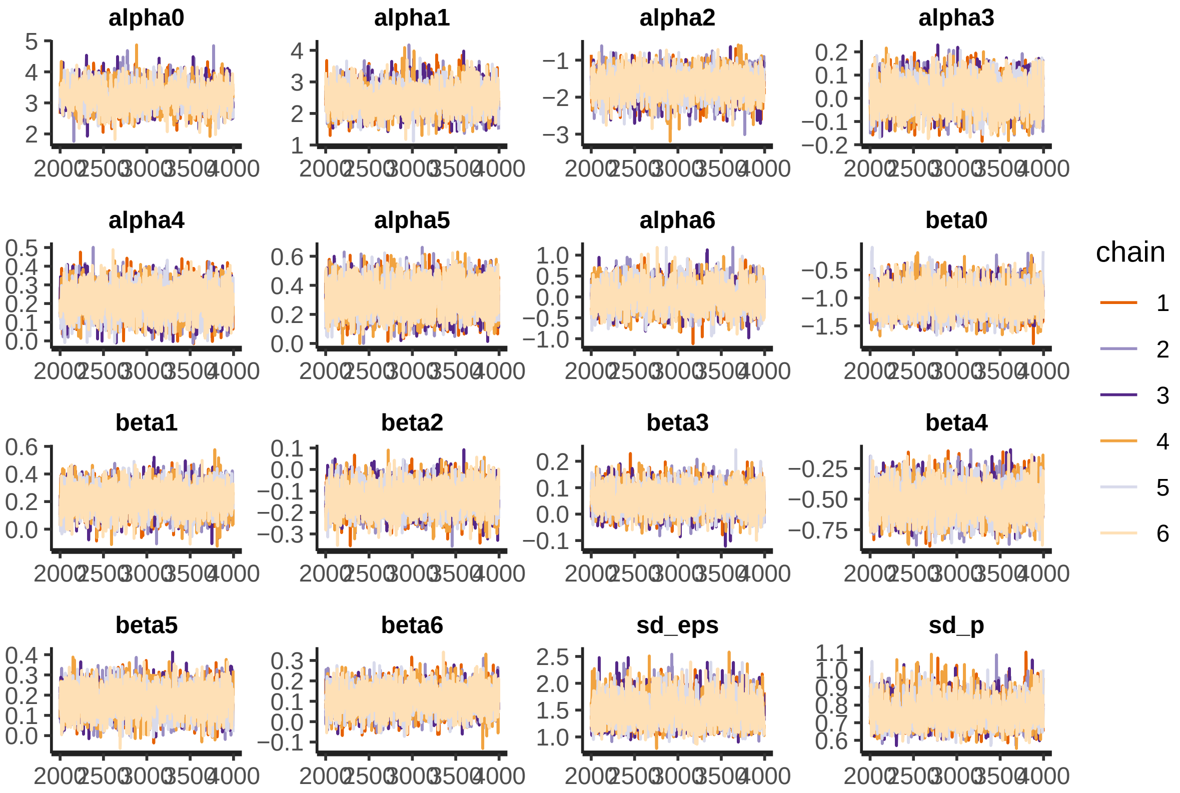

Check Domain Specific Expectations

Check N for Truncation

The augmentation to marginalize N out as a latent discrete requires setting an upper bound, K, to loop through. If K is too small the posterior will be truncated. Need to check for every N.

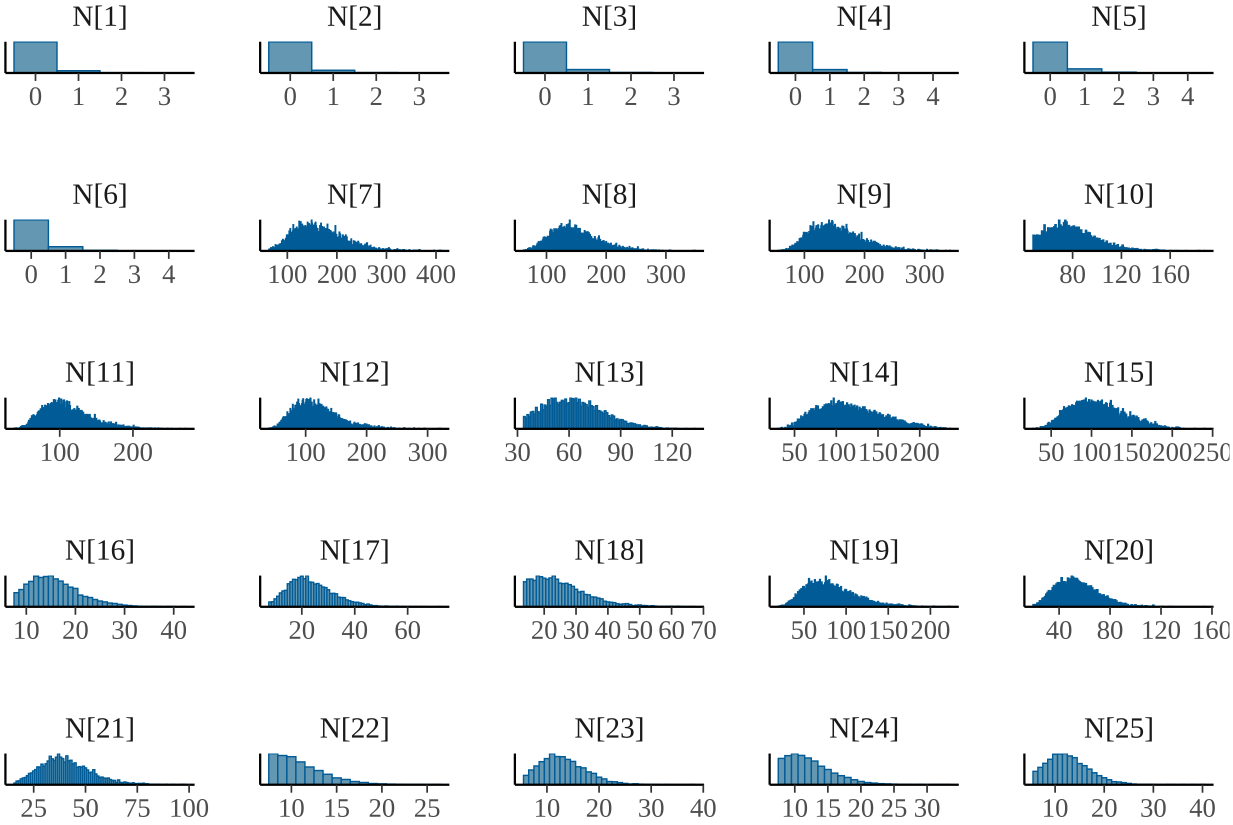
N[1]
0 1 2 3
N[2]
0 1 2 3
N[3]
0 1 2 3
N[4]
0 1 2 3 4
N[5]
0 1 2 3 4
N[6]
0 1 2 3 4
N[7]
100 200 300 400
N[8]
100 200 300
N[9]
100 200 300
N[10]
80 120 160
N[11]
100 200
N[12]
100 200 300
N[13]
30 60 90 120
N[14]
50 100 150 200
N[15]
50 100 150 200 250
N[16]
10 20 30 40
N[17]
20 40 60
N[18]
20 30 40 50 60 70
N[19]
50 100 150 200
N[20]
40 80 120 160
N[21]
25 50 75 100
N[22]
10 15 20 25
N[23]
10 20 30 40
N[24]
10 15 20 25 30
N[25]
10 20 30 40

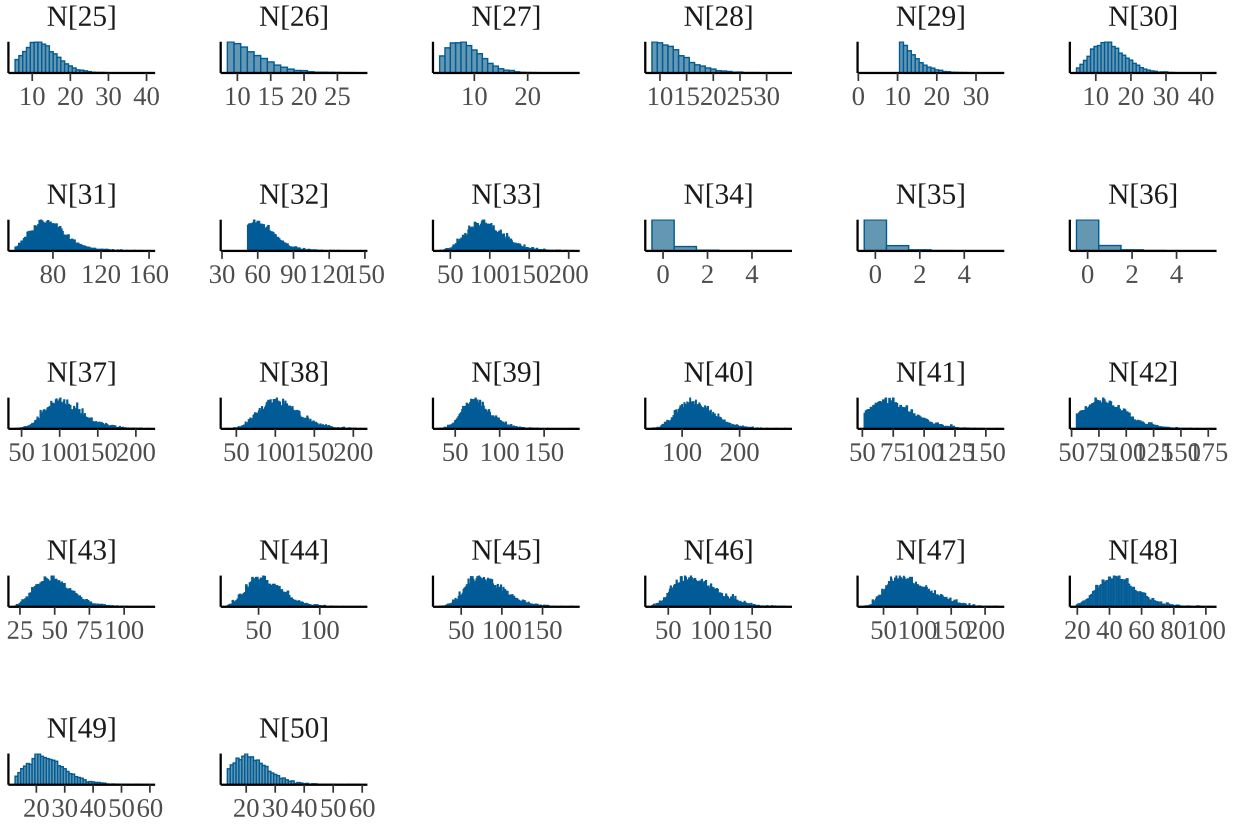
N[25]
10 20 30 40
N[26]
10 15 20 25
N[27]
10 20
N[28]
1015202530
N[29]
0 10 20 30
N[30]
10 20 30 40
N[31]
80 120 160
N[32]
30 60 90 120150
N[33]
50 100150200
N[34]
0 2 4
N[35]
0 2 4
N[36]
0 2 4
N[37]
50 100150200
N[38]
50 100150200
N[39]
50 100 150
N[40]
100 200
N[41]
50 75100125150
N[42]
5075100125150175
N[43]
25 50 75100
N[44]
50 100
N[45]
50 100150
N[46]
50 100 150
N[47]
50100150200
N[48]
20 40 60 80100
N[49]
2030405060
N[50]
2030405060

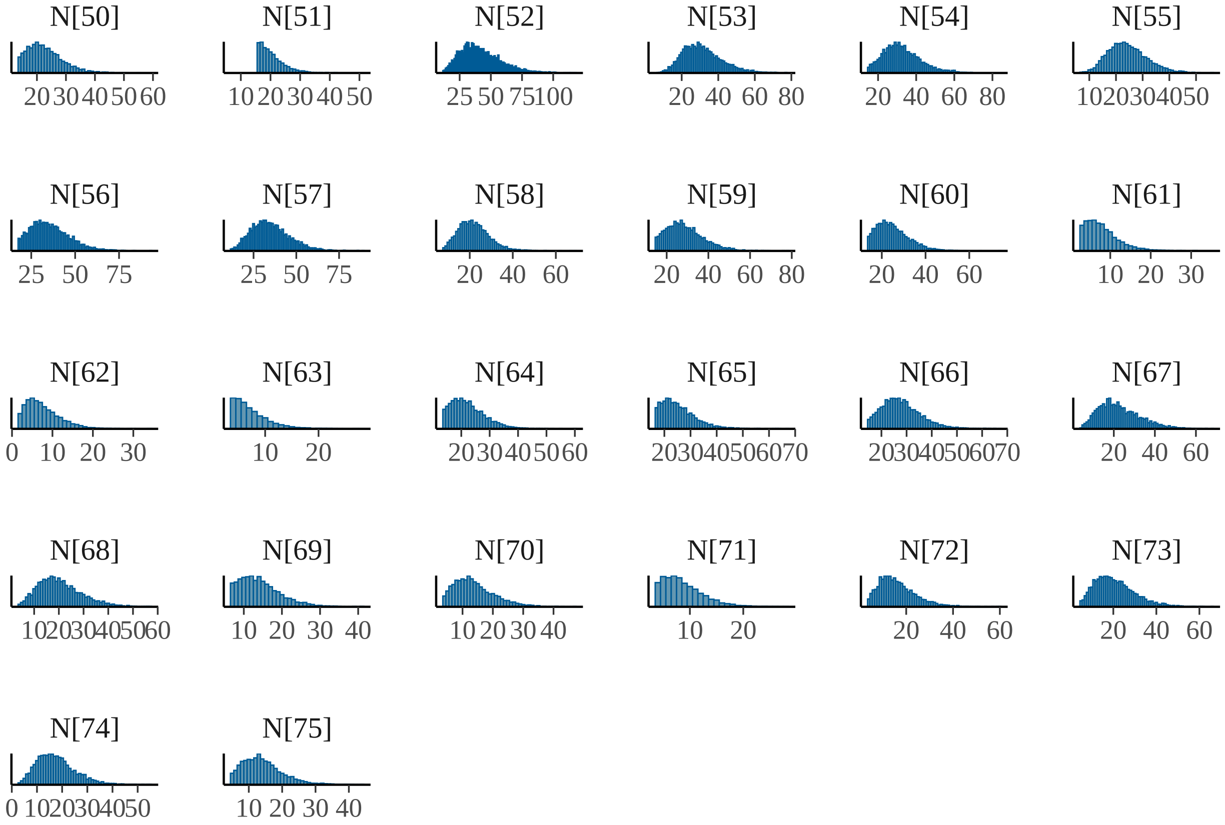
N[50]
20 30 40 50 60
N[51]
10 20 30 40 50
N[52]
25 50 75 100
N[53]
20 40 60 80
N[54]
20 40 60 80
N[55]
10 20 30 40 50
N[56]
25 50 75
N[57]
25 50 75
N[58]
20 40 60
N[59]
20 40 60 80
N[60]
20 40 60
N[61]
10 20 30
N[62]
0 10 20 30
N[63]
10 20
N[64]
20 30 40 50 60
N[65]
20 30 40 50 60 70
N[66]
20 30 40 50 60 70
N[67]
20 40 60
N[68]
10 20 30 40 50 60
N[69]
10 20 30 40
N[70]
10 20 30 40
N[71]
10 20
N[72]
20 40 60
N[73]
20 40 60
N[74]
0 10 20 30 40 50
N[75]
10 20 30 40

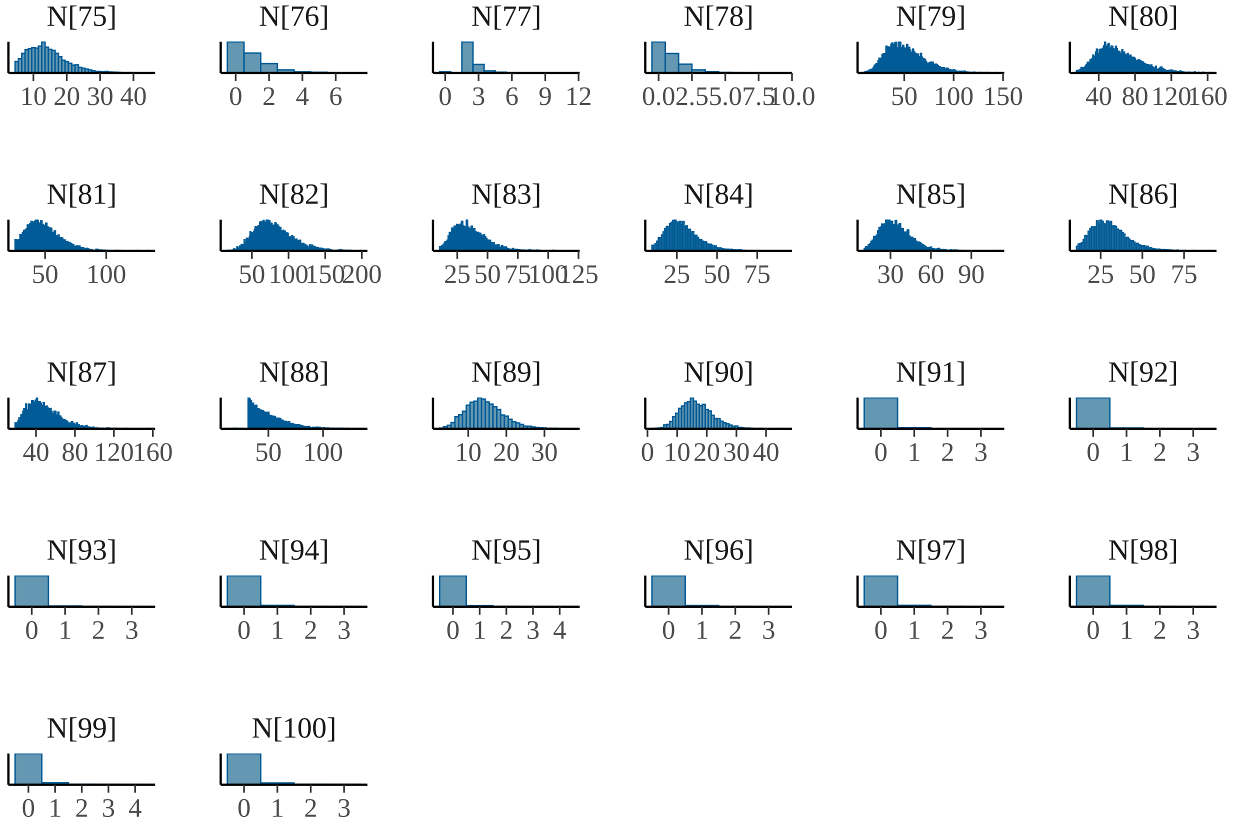
N[75]
10 20 30 40
N[76]
0 2 4 6
N[77]
0 3 6 9 12
N[78]
0.02.55.07.510.0
N[79]
50 100 150
N[80]
40 80 120160
N[81]
50 100
N[82]
50 100150200
N[83]
25 50 75100125
N[84]
25 50 75
N[85]
30 60 90
N[86]
25 50 75
N[87]
40 80 120160
N[88]
50 100
N[89]
10 20 30
N[90]
0 10 20 30 40
N[91]
0 1 2 3
N[92]
0 1 2 3
N[93]
0 1 2 3
N[94]
0 1 2 3
N[95]
0 1 2 3 4
N[96]
0 1 2 3
N[97]
0 1 2 3
N[98]
0 1 2 3
N[99]
0 1 2 3 4
N[100]
0 1 2 3

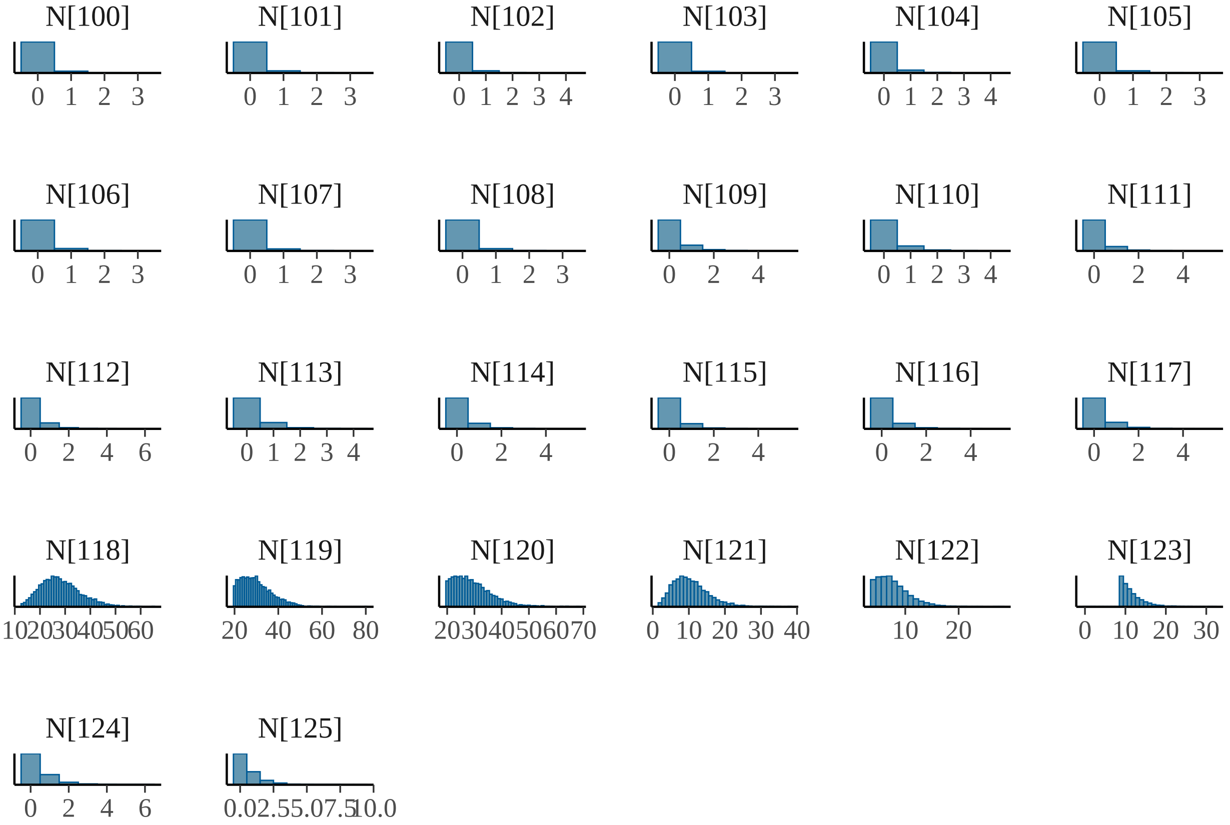
N[100]
N[101]
N[102]
N[103]
N[104]
N[105]
N[106]
N[107]
N[108]
N[109]
N[110]
N[111]
N[112]
N[113]
N[114]
N[115]
N[116]
N[117]
N[118]
N[119]
N[120]
N[121]
N[122]
N[123]
N[124]
N[125]

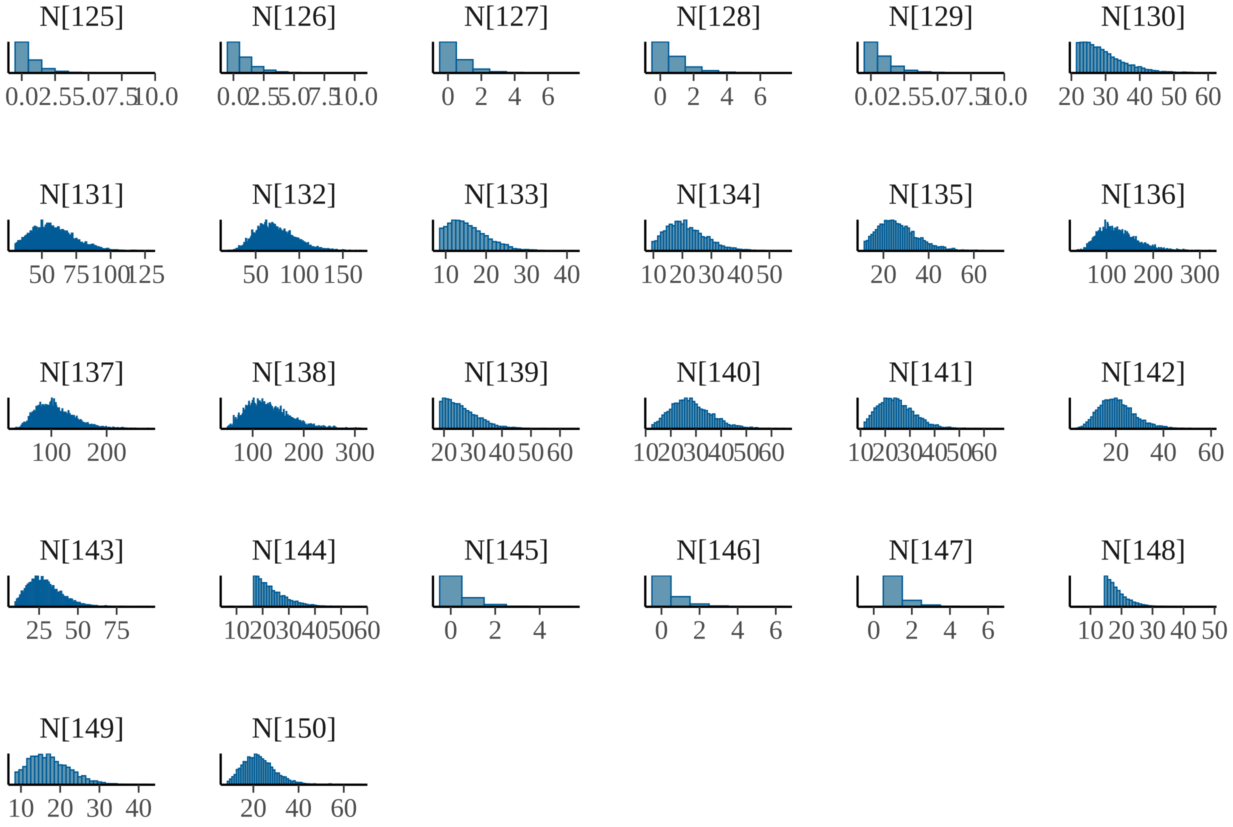
N[125]
N[126]
N[127]
N[128]
N[129]
N[130]
N[131]
N[132]
N[133]
N[134]
N[135]
N[136]
N[137]
N[138]
N[139]
N[140]
N[141]
N[142]
N[143]
N[144]
N[145]
N[146]
N[147]
N[148]
N[149]
N[150]

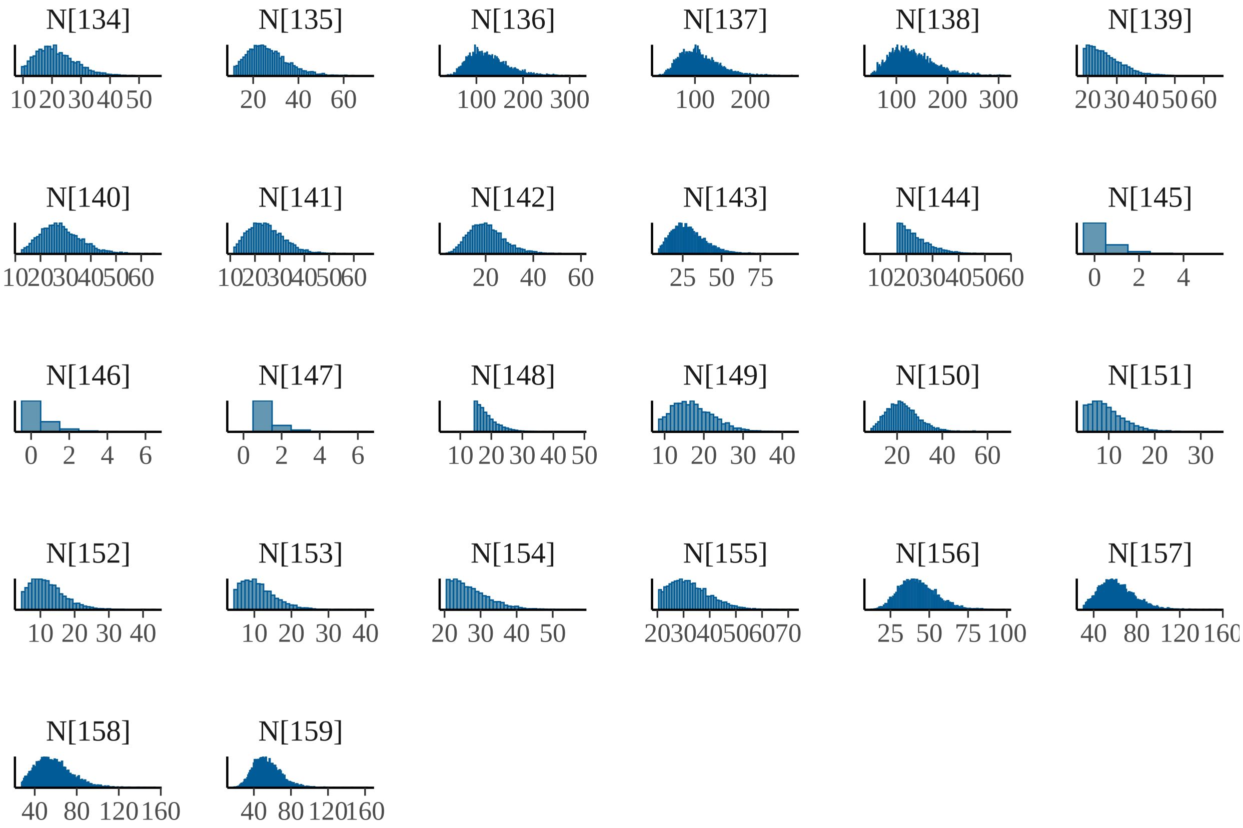


```
## null device
##           1
```

Check detection

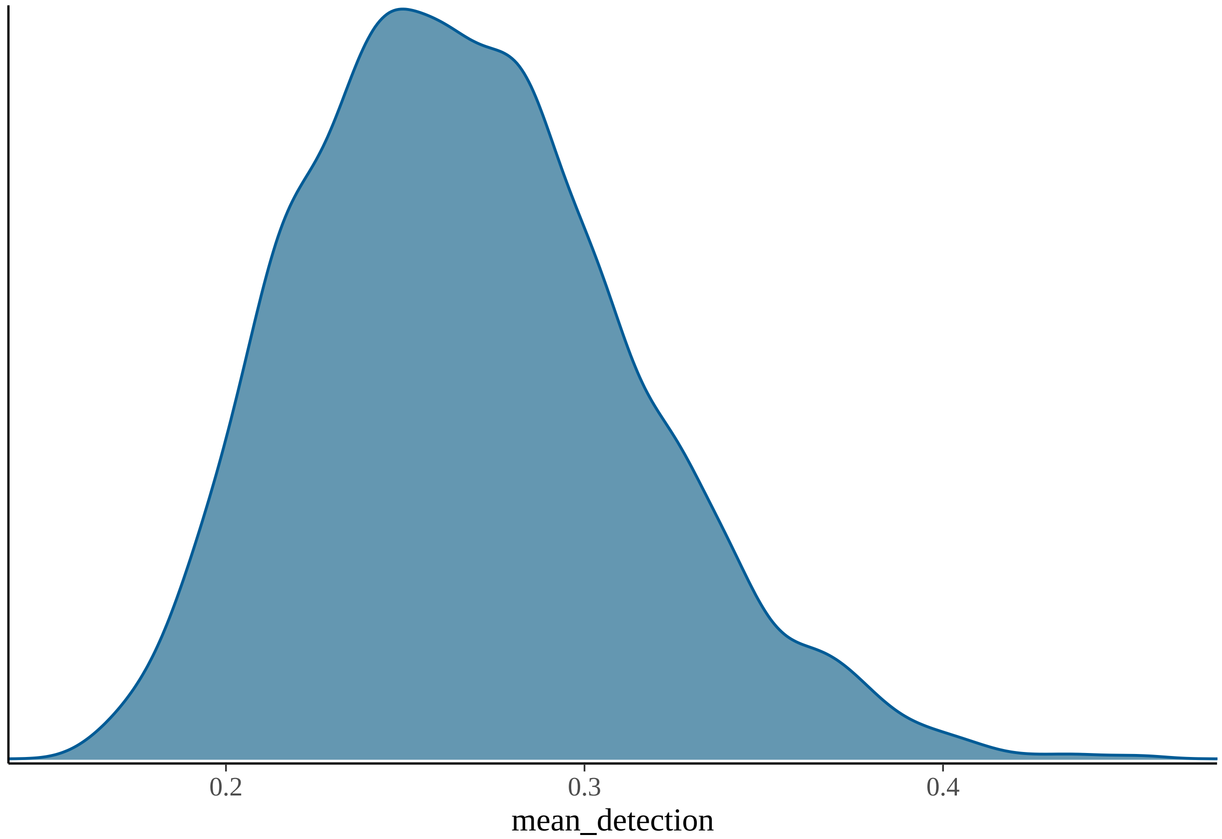

Check Divergences and Pairwise Correlations

```
## 0 of 6000 iterations ended with a divergence.
```

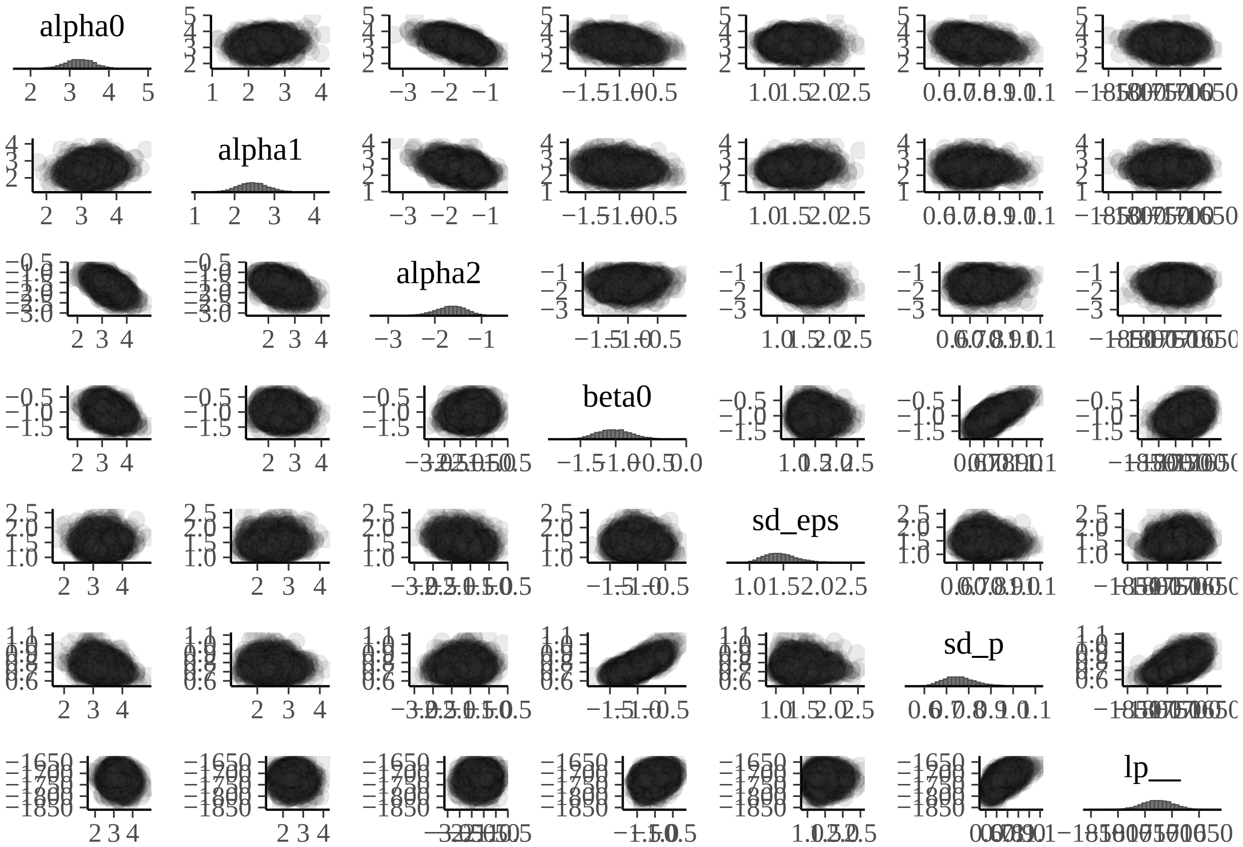

Check Energy and Treedepth

```
## E-BFMI indicated no pathological behavior.

##

## 0 of 6000 iterations saturated the maximum tree depth of 10.

##
```

Summarize Samples Sizes and Mixing

Effective samples sizes using rstan::monitor following Hoffman and Gelman (2014) to be more reliable and accurate (as in Monnahan et al. 2017) - UPDATE - now follow Vehtari et al. 2019.

effective sample sizes (should be > 100) - Aki Vehtari, Andrew Gelman, Daniel Simpson, Bob Carpenter, and Paul-Christian Bürkner (2019). Rank-normalization, folding, and localization: An improved R-hat for assessing convergence of MCMC. arXiv preprint arXiv:1903.08008.

```
## [1] 1.003609

## [1] 1675

## [1] 2564
```

Posterior Predictive Checks

Examine posterior prredictions of total counts across all 5 visits

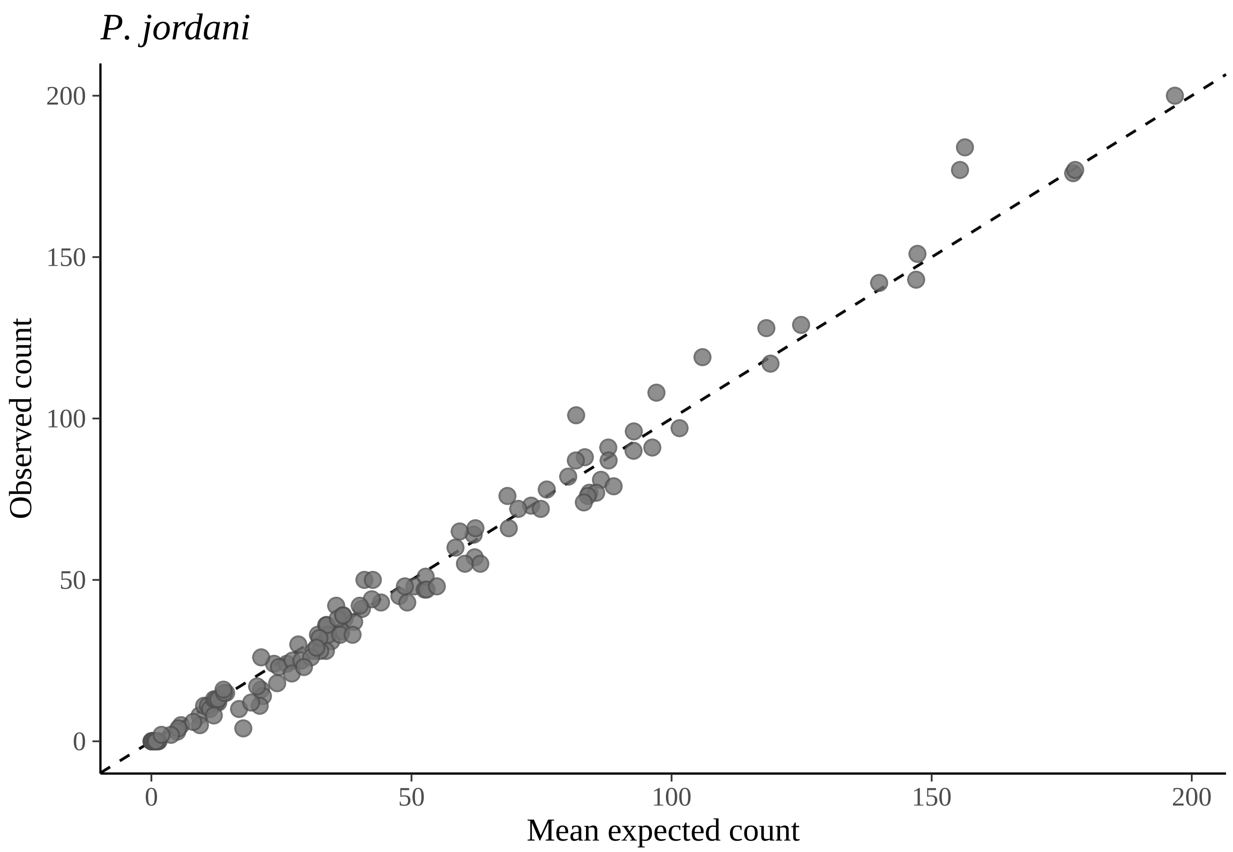

RMSE of posterior predictive

```
## [1] 5.070722
```

Posterior predictive check for each visit

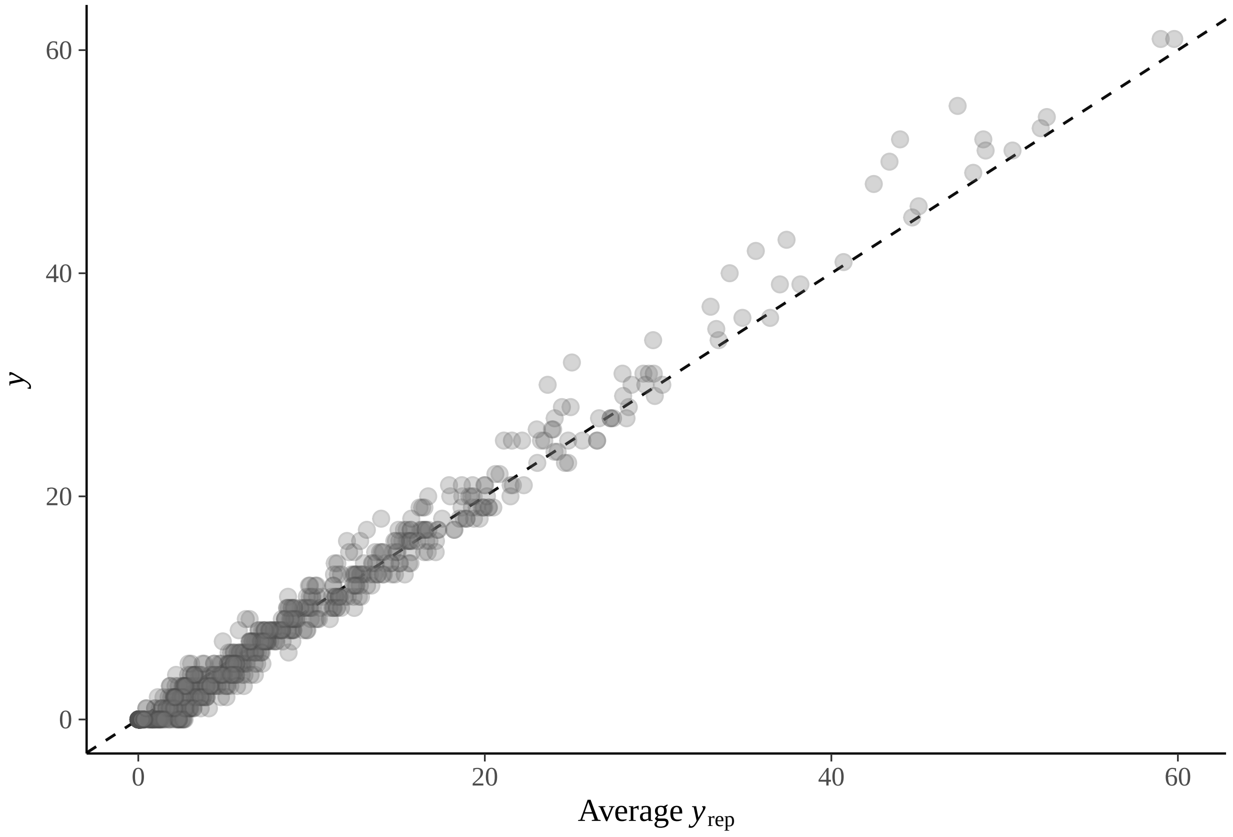
60
40
20
0
y
0
20
40
60
Average y_{rep}

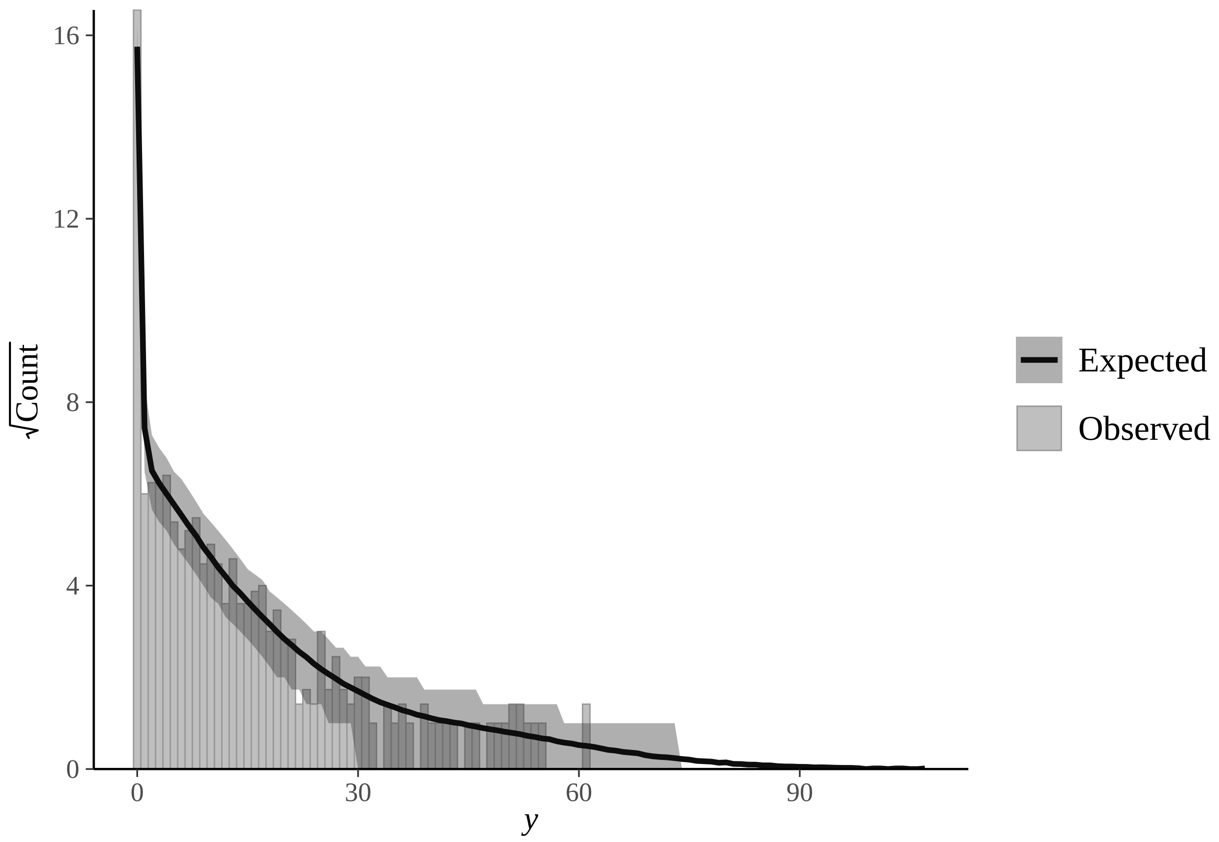
√Count
16
12
8
4
0
0
30
60
90
y
Expected
Observed

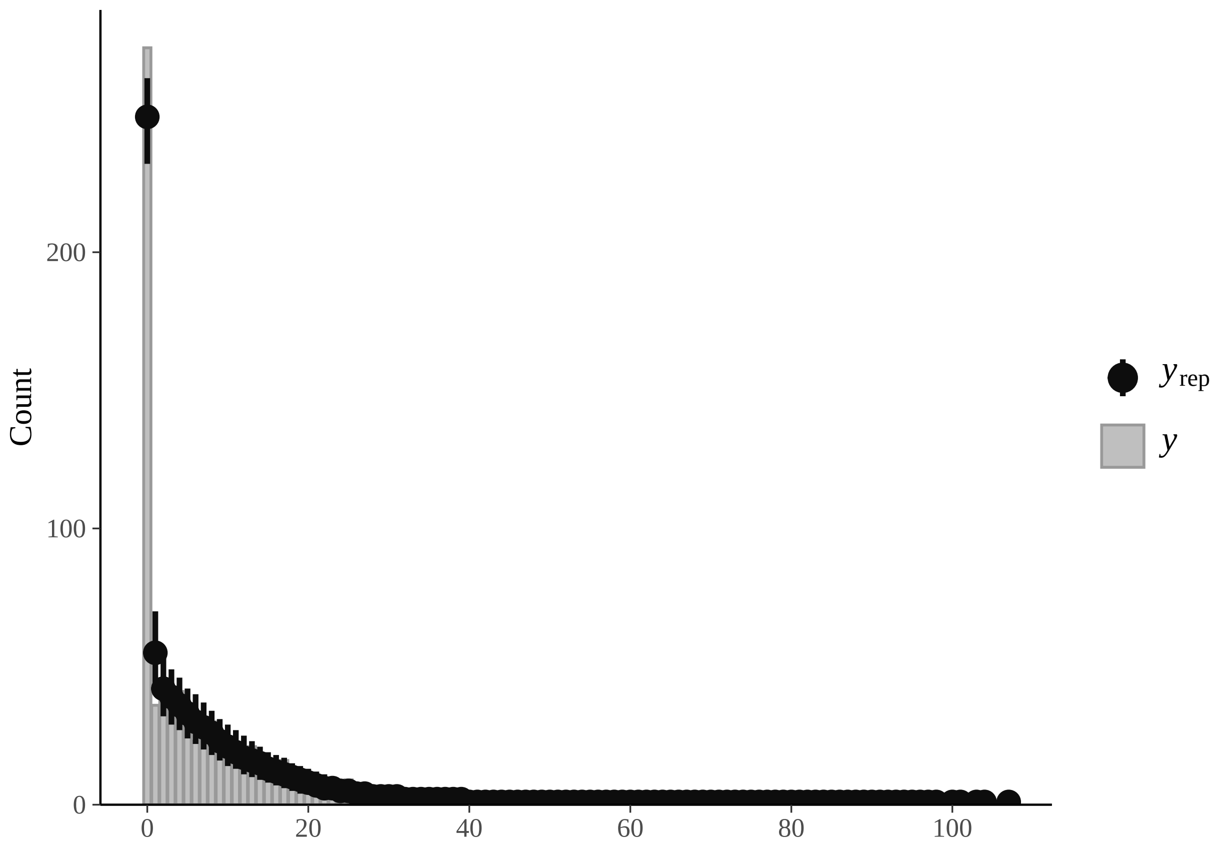

Count
200
100
0
0
20
40
60
80
100
y_{rep}
y

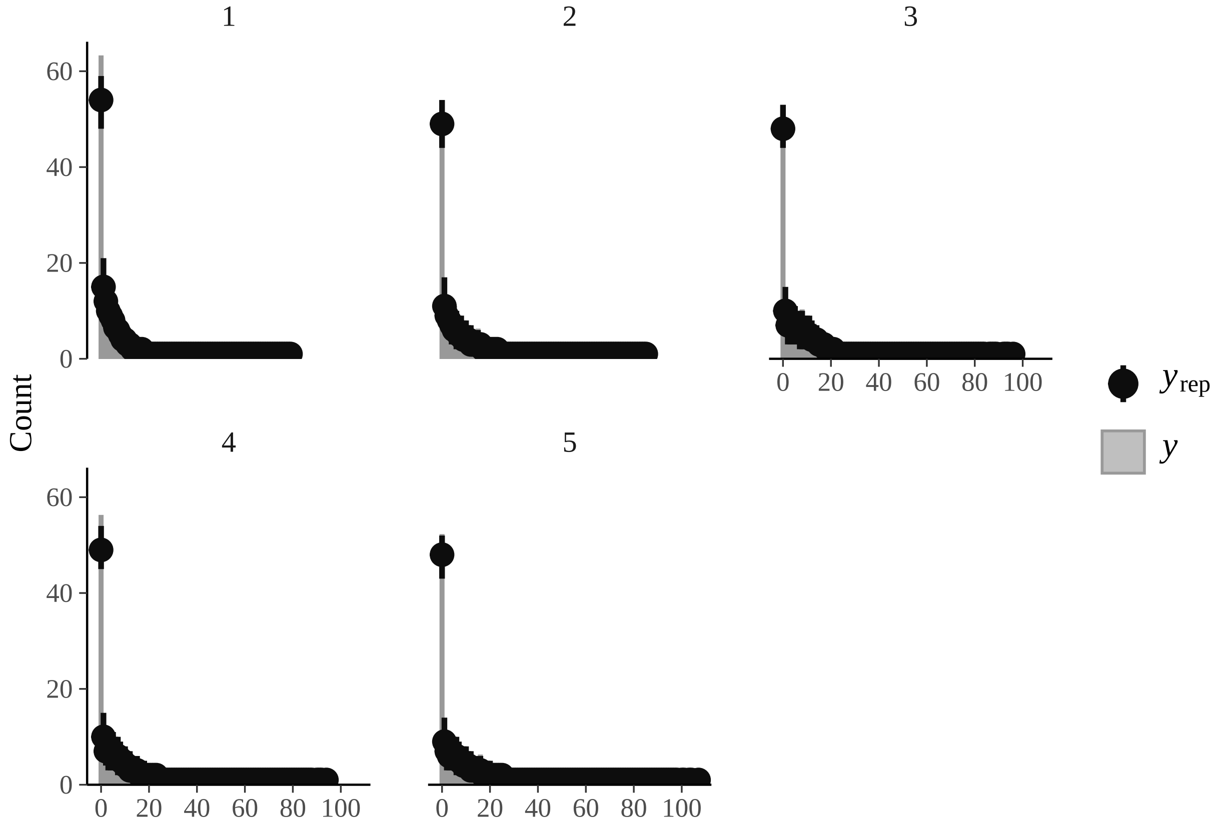

RMSE of posterior predictive for observations per visit

```
## [1] 2.956443
```

RMSE of posterior predictive

```
## [1] 1.322162
```

Desmognathus wrighti

View Traceplots

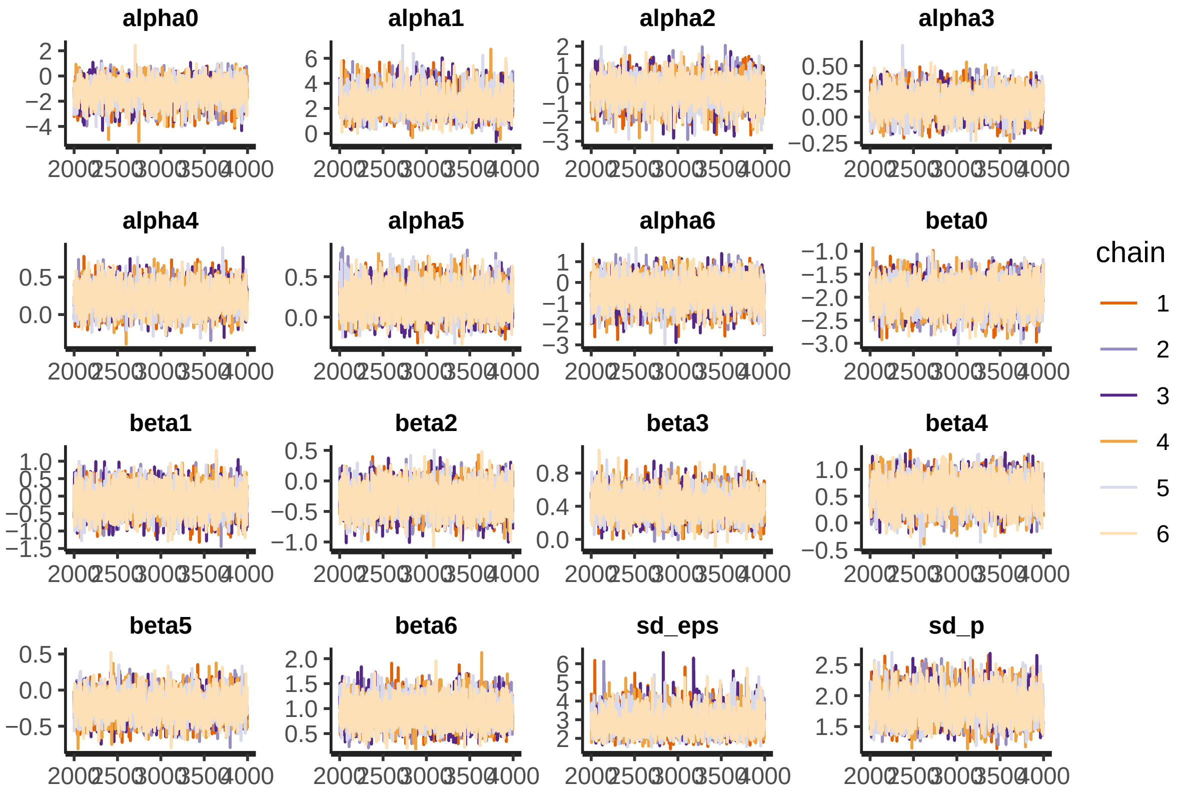

Check Domain Specific Expectations

Check N for Truncation

The augmentation to marginalize N out as a latent discrete requires setting an upper bound, K, to loop through. If K is too small the posterior will be truncated. Need to check for every N.

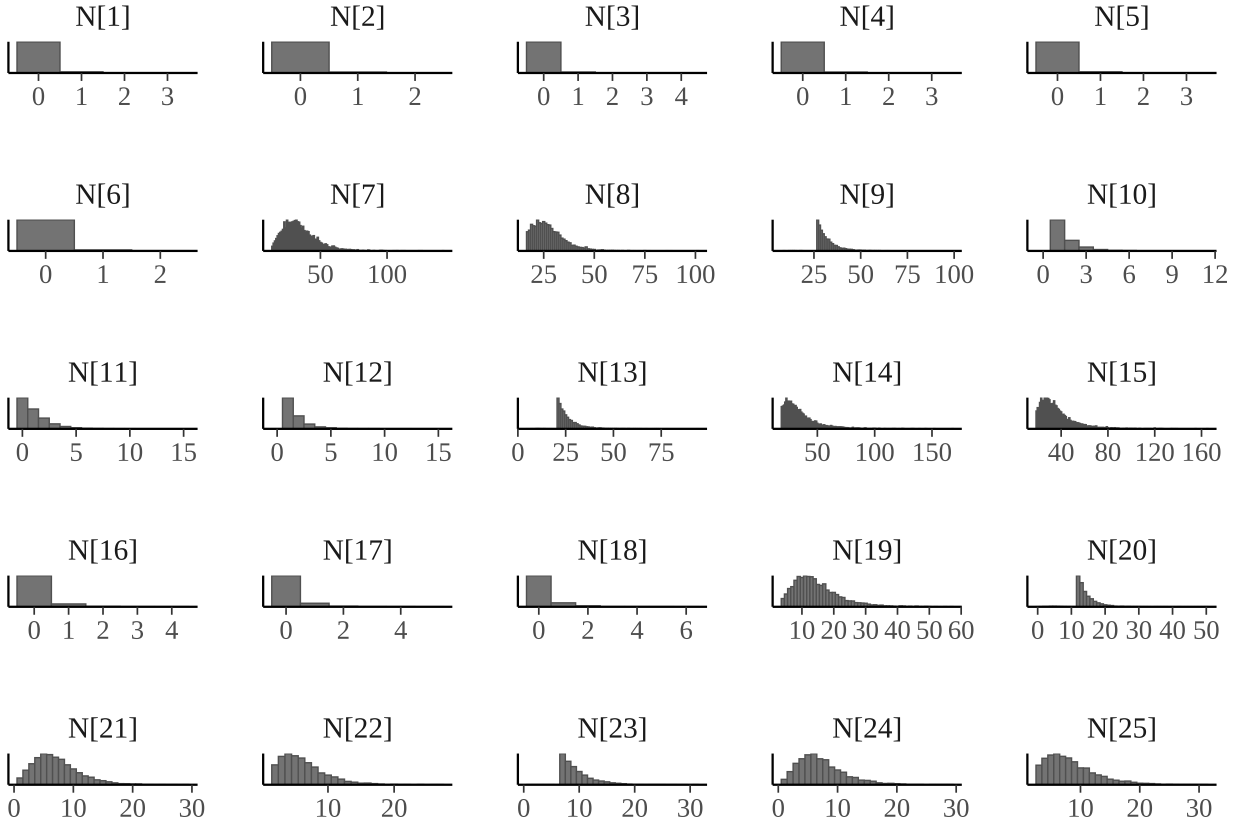
N[1]
0 1 2 3
N[2]
0 1 2
N[3]
0 1 2 3 4
N[4]
0 1 2 3
N[5]
0 1 2 3
N[6]
0 1 2
N[7]
50 100
N[8]
25 50 75 100
N[9]
25 50 75 100
N[10]
0 3 6 9 12
N[11]
0 5 10 15
N[12]
0 5 10 15
N[13]
0 25 50 75
N[14]
50 100 150
N[15]
40 80 120 160
N[16]
0 1 2 3 4
N[17]
0 2 4
N[18]
0 2 4 6
N[19]
10 20 30 40 50 60
N[20]
0 10 20 30 40 50
N[21]
0 10 20 30
N[22]
10 20
N[23]
0 10 20 30
N[24]
0 10 20 30
N[25]
10 20 30

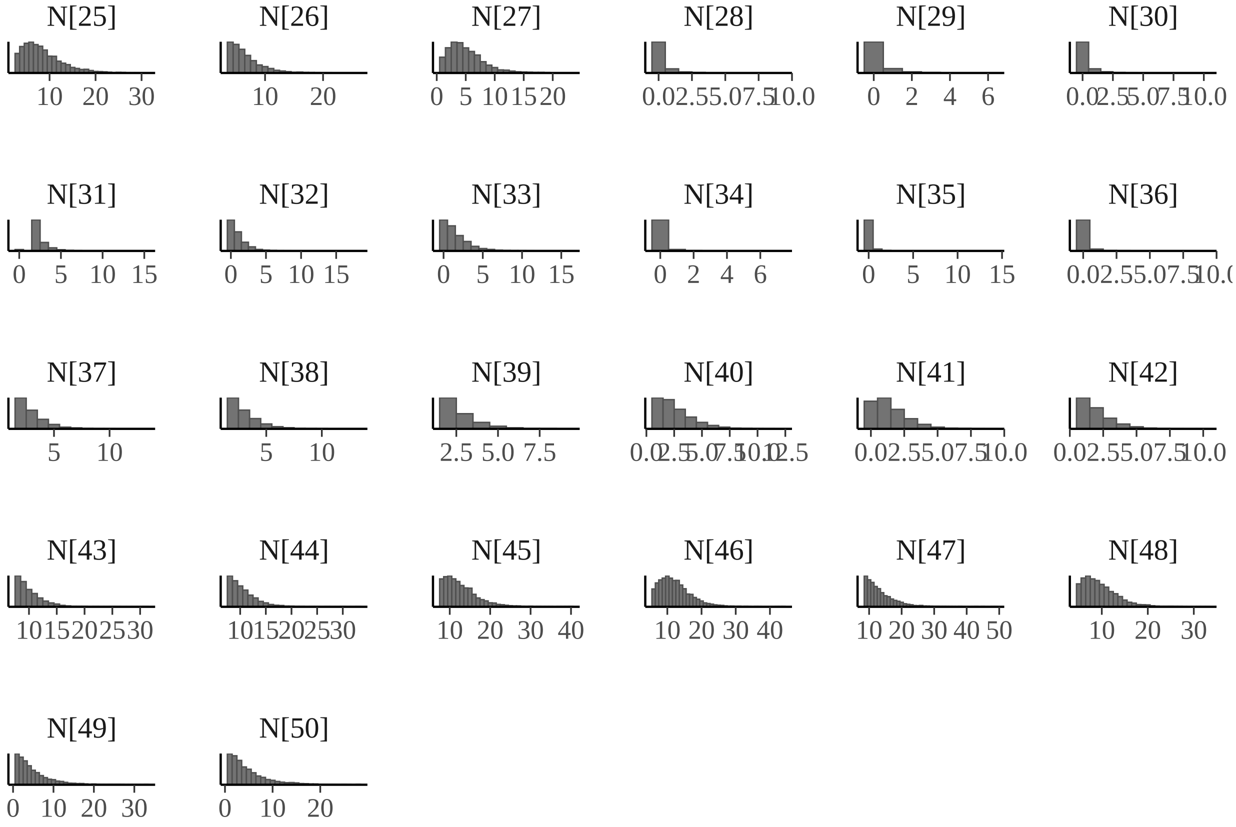
N[25]
N[26]
N[27]
N[28]
N[29]
N[30]
N[31]
N[32]
N[33]
N[34]
N[35]
N[36]
N[37]
N[38]
N[39]
N[40]
N[41]
N[42]
N[43]
N[44]
N[45]
N[46]
N[47]
N[48]
N[49]
N[50]

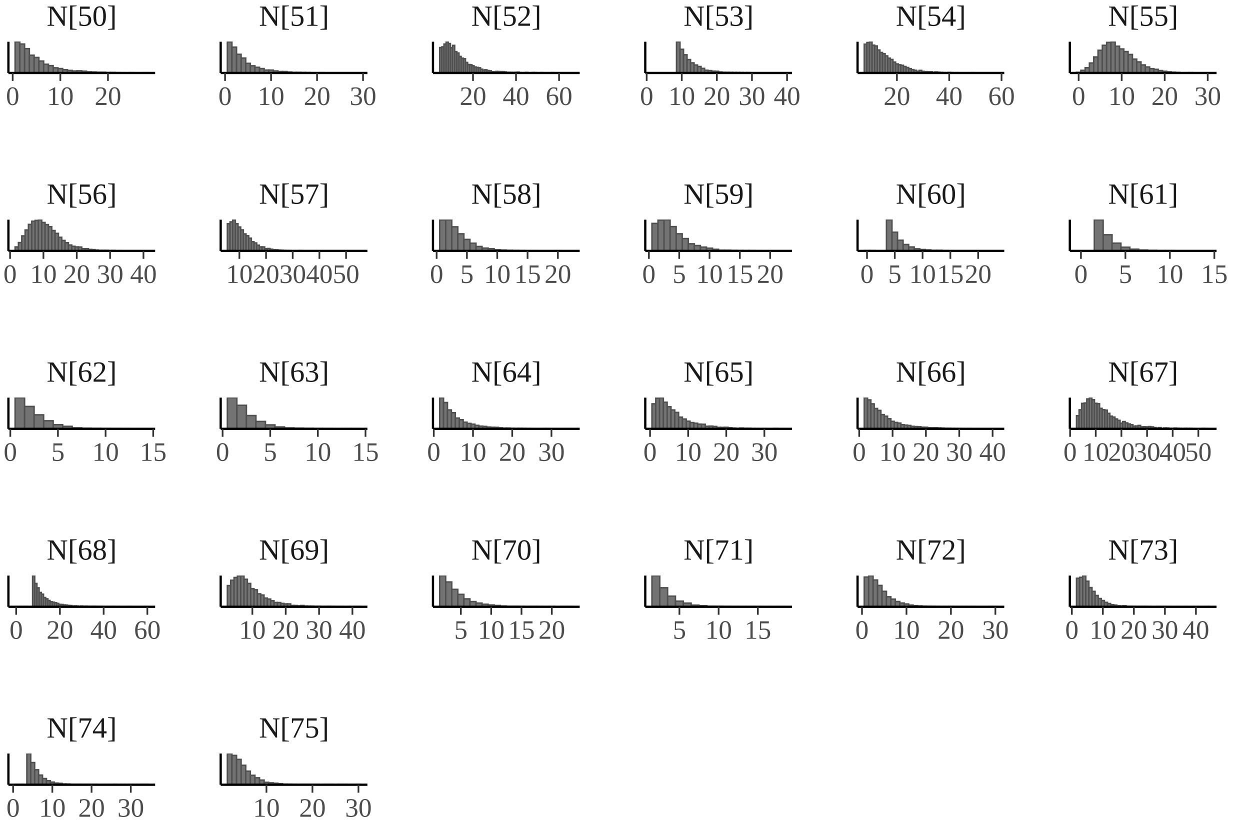
N[50]
0 10 20
N[51]
0 10 20 30
N[52]
20 40 60
N[53]
0 10 20 30 40
N[54]
20 40 60
N[55]
0 10 20 30
N[56]
0 10 20 30 40
N[57]
1020304050
N[58]
0 5 10 15 20
N[59]
0 5 10 15 20
N[60]
0 5 101520
N[61]
0 5 10 15
N[62]
0 5 10 15
N[63]
0 5 10 15
N[64]
0 10 20 30
N[65]
0 10 20 30
N[66]
0 10 20 30 40
N[67]
0 1020304050
N[68]
0 20 40 60
N[69]
10 20 30 40
N[70]
5 10 15 20
N[71]
5 10 15
N[72]
0 10 20 30
N[73]
0 10 20 30 40
N[74]
0 10 20 30
N[75]
10 20 30

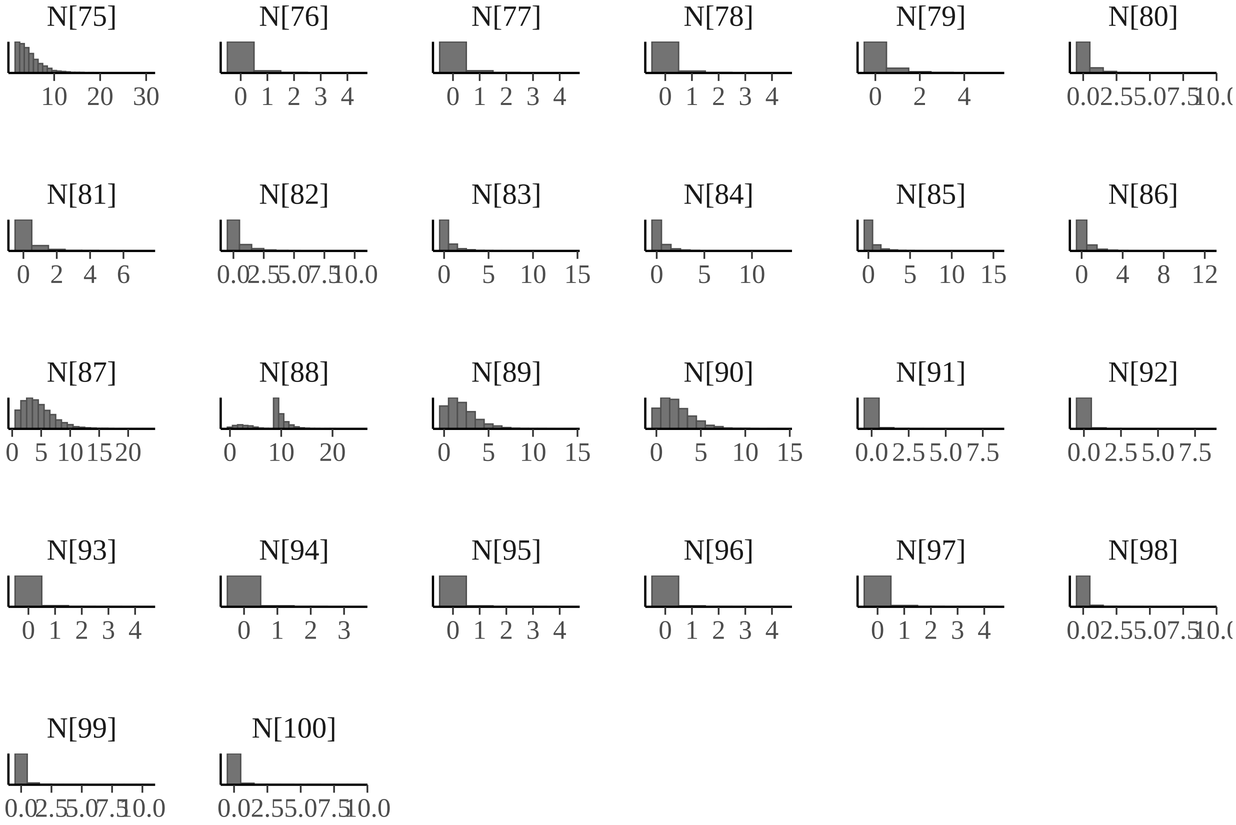
N[75]
10 20 30
N[76]
0 1 2 3 4
N[77]
0 1 2 3 4
N[78]
0 1 2 3 4
N[79]
0 2 4
N[80]
N[81]
0 2 4 6
N[82]
N[83]
0 5 10 15
N[84]
0 5 10
N[85]
0 5 10 15
N[86]
0 4 8 12
N[87]
0 5 10 15 20
N[88]
0 10 20
N[89]
0 5 10 15
N[90]
0 5 10 15
N[91]
0.0 2.5 5.0 7.5
N[92]
0.0 2.5 5.0 7.5
N[93]
0 1 2 3 4
N[94]
0 1 2 3
N[95]
0 1 2 3 4
N[96]
0 1 2 3 4
N[97]
0 1 2 3 4
N[98]
N[99]
N[100]

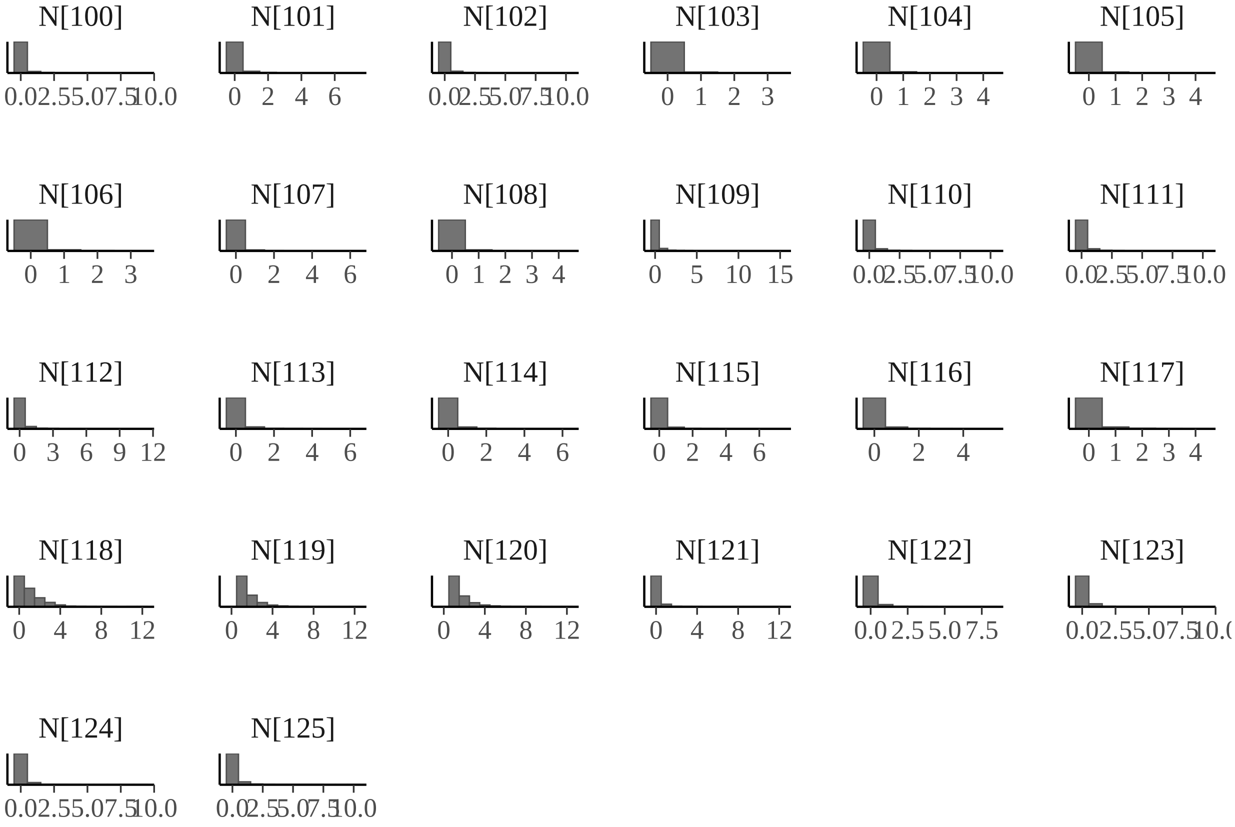
N[100]
0.02.55.07.510.0
N[101]
0 2 4 6
N[102]
0.02.55.07.510.0
N[103]
0 1 2 3
N[104]
0 1 2 3 4
N[105]
0 1 2 3 4
N[106]
0 1 2 3
N[107]
0 2 4 6
N[108]
0 1 2 3 4
N[109]
0 5 10 15
N[110]
0.02.55.07.510.0
N[111]
0.02.55.07.510.0
N[112]
0 3 6 9 12
N[113]
0 2 4 6
N[114]
0 2 4 6
N[115]
0 2 4 6
N[116]
0 2 4
N[117]
0 1 2 3 4
N[118]
0 4 8 12
N[119]
0 4 8 12
N[120]
0 4 8 12
N[121]
0 4 8 12
N[122]
0.0 2.5 5.0 7.5
N[123]
0.02.55.07.510.0
N[124]
0.02.55.07.510.0
N[125]
0.02.55.07.510.0

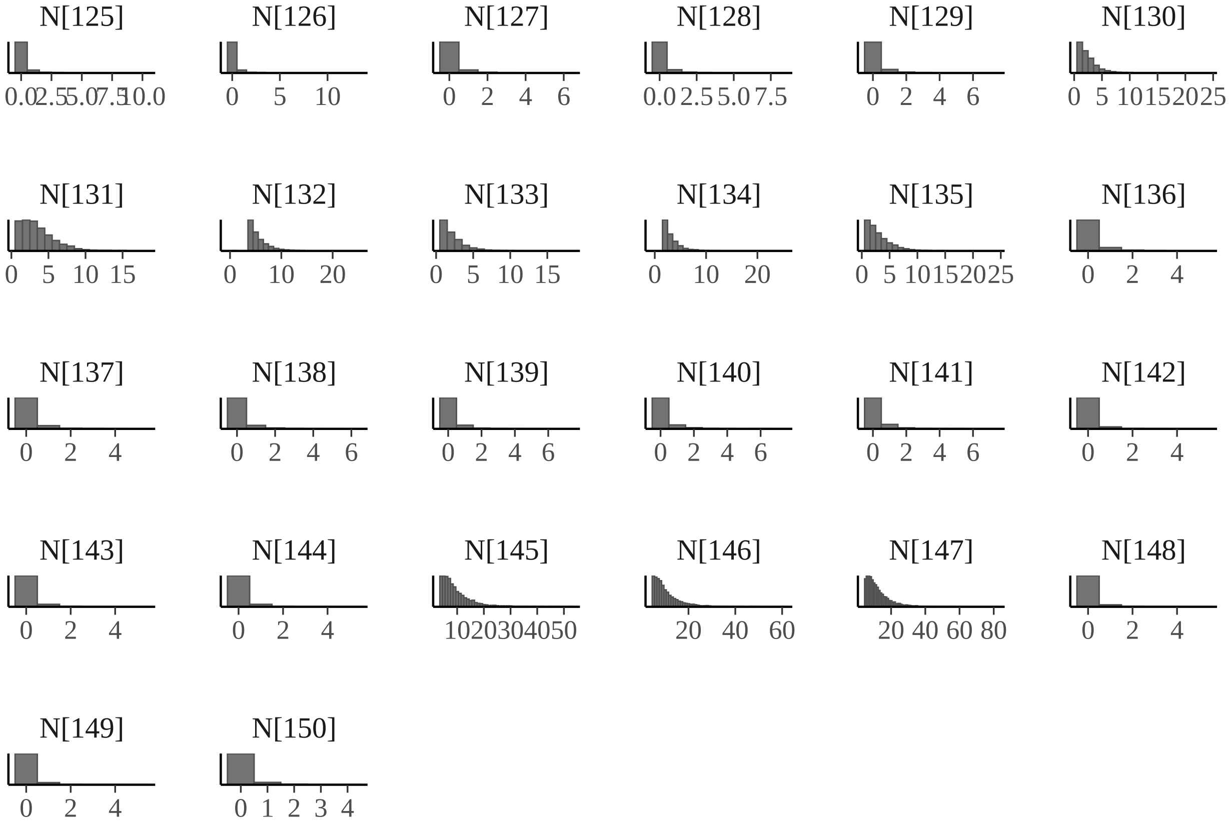
N[125]
N[126]
N[127]
N[128]
N[129]
N[130]
N[131]
N[132]
N[133]
N[134]
N[135]
N[136]
N[137]
N[138]
N[139]
N[140]
N[141]
N[142]
N[143]
N[144]
N[145]
N[146]
N[147]
N[148]
N[149]
N[150]

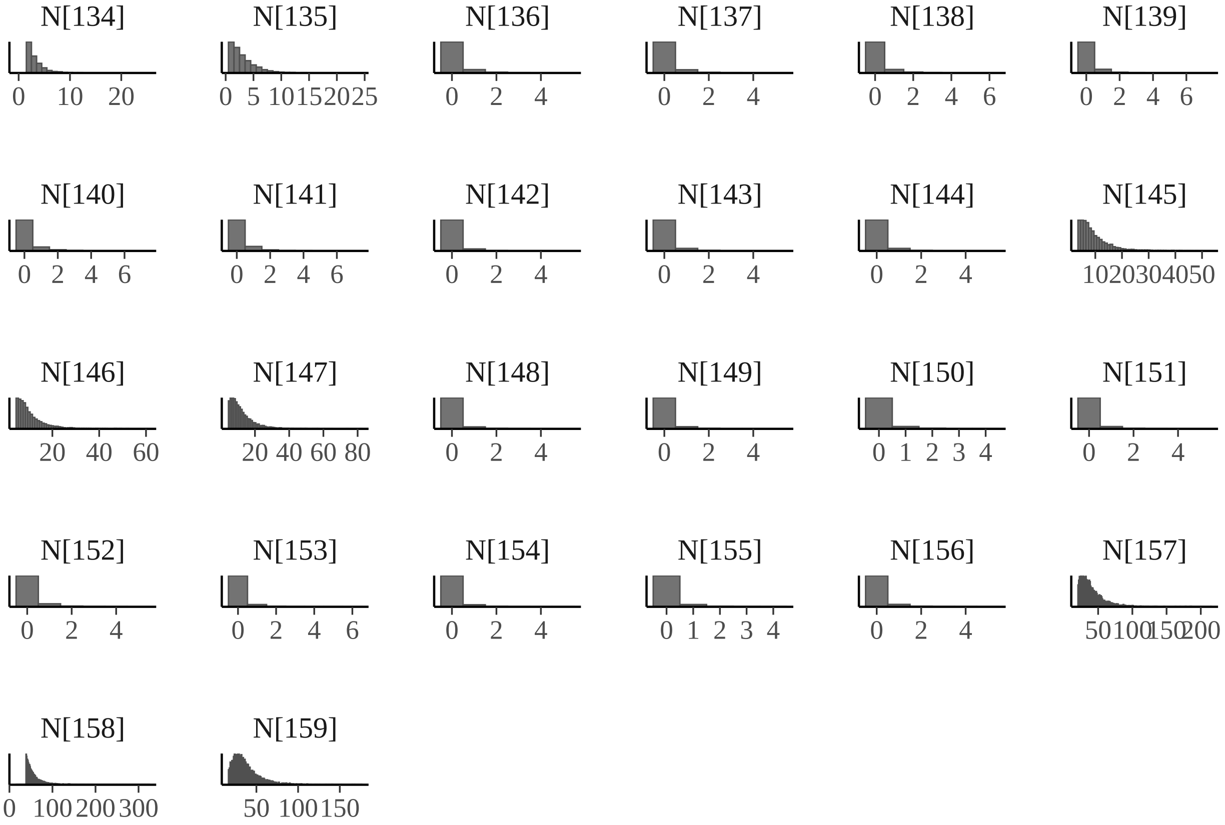


```
## null device
##           1
```

Check detection

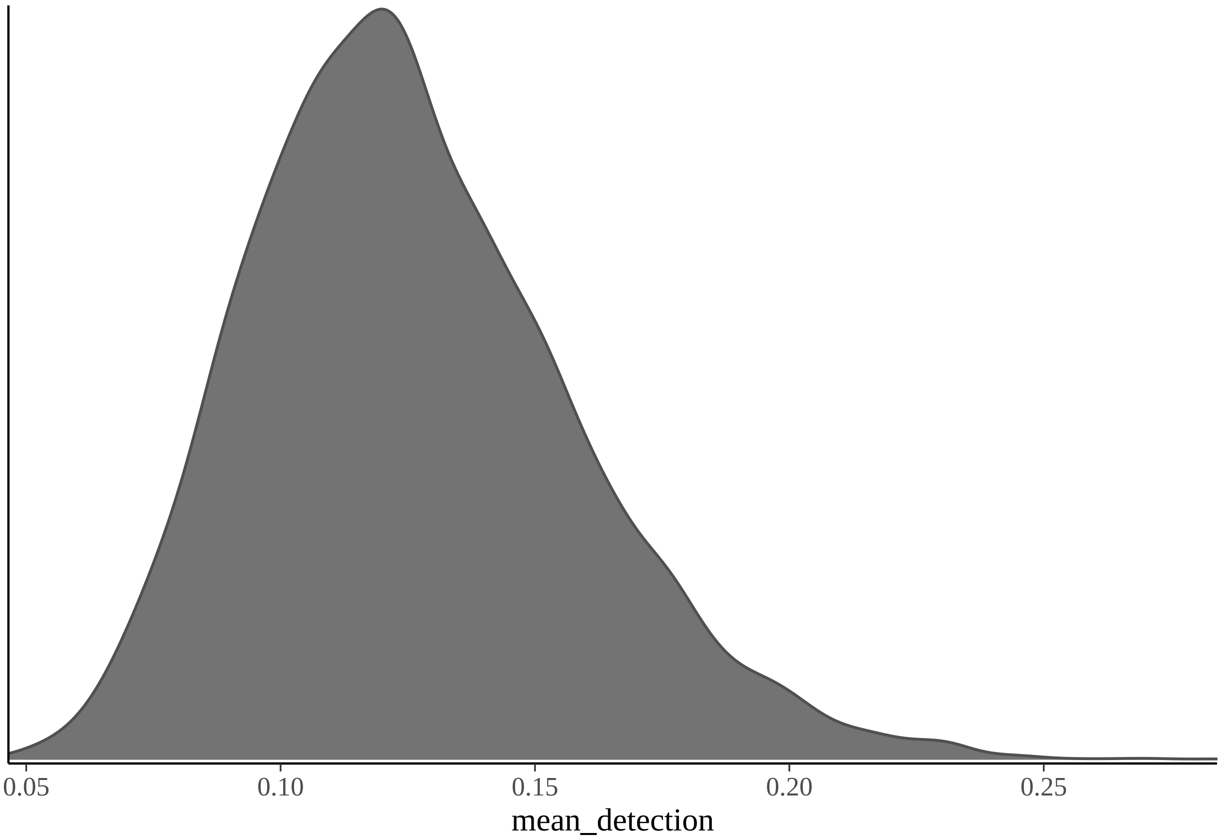

Check Divergences and Pairwise Correlations

```
## 0 of 6000 iterations ended with a divergence.

##
```

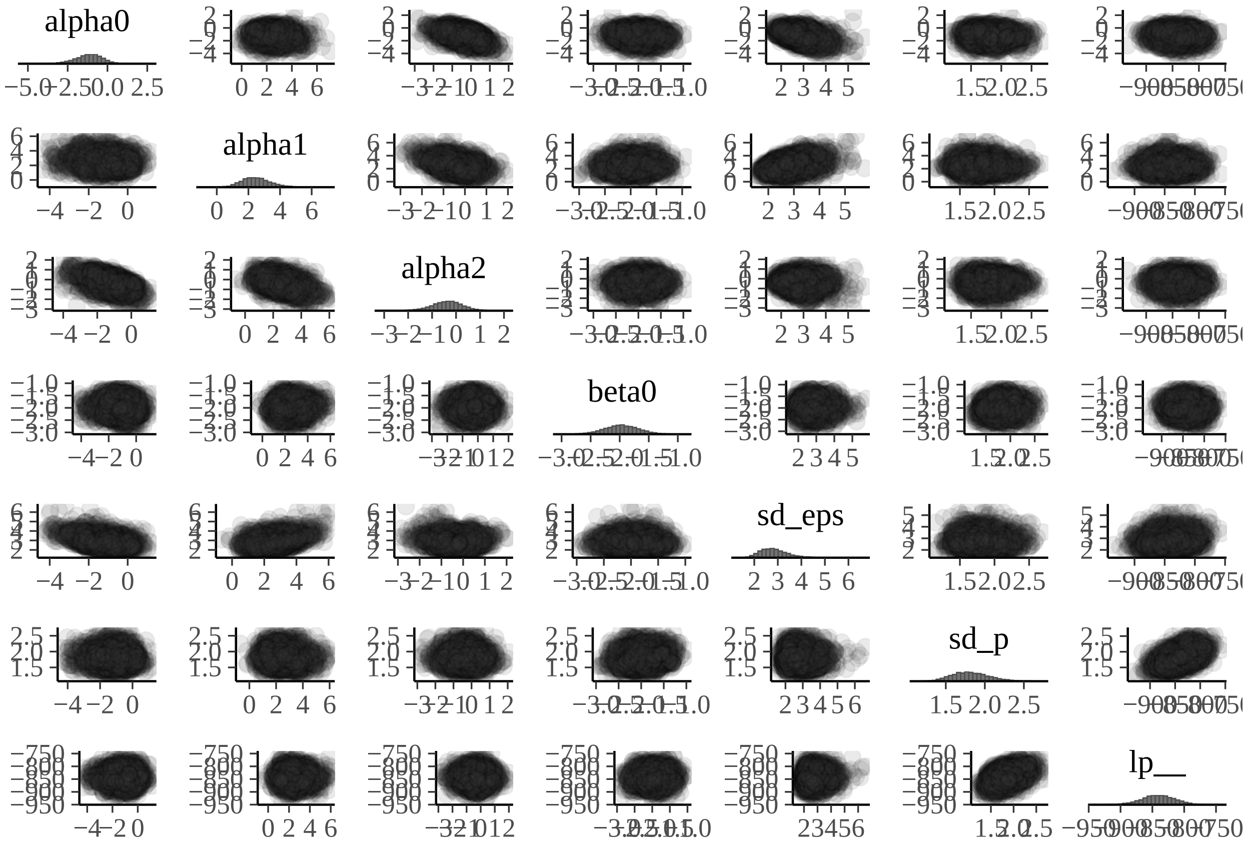

Check Energy and Treedepth

```
## E-BFMI indicated no pathological behavior.

##

## 0 of 6000 iterations saturated the maximum tree depth of 10.

##
```

Summarize Samples Sizes and Mixing

Effective samples sizes using rstan::monitor following Hoffman and Gelman (2014) to be more reliable and accurate (as in Monnahan et al. 2017) - UPDATE - now follow Vehtari et al. 2019.

effective sample sizes (should be > 100) - Aki Vehtari, Andrew Gelman, Daniel Simpson, Bob Carpenter, and Paul-Christian Bürkner (2019). Rank-normalization, folding, and localization: An improved R-hat for assessing convergence of MCMC. arXiv preprint arXiv:1903.08008.

```
## [1] 1.00399

## [1] 2346

## [1] 2987
```

Posterior Predictive Checks

Examine posterior prredictions of total counts across all 5 visits

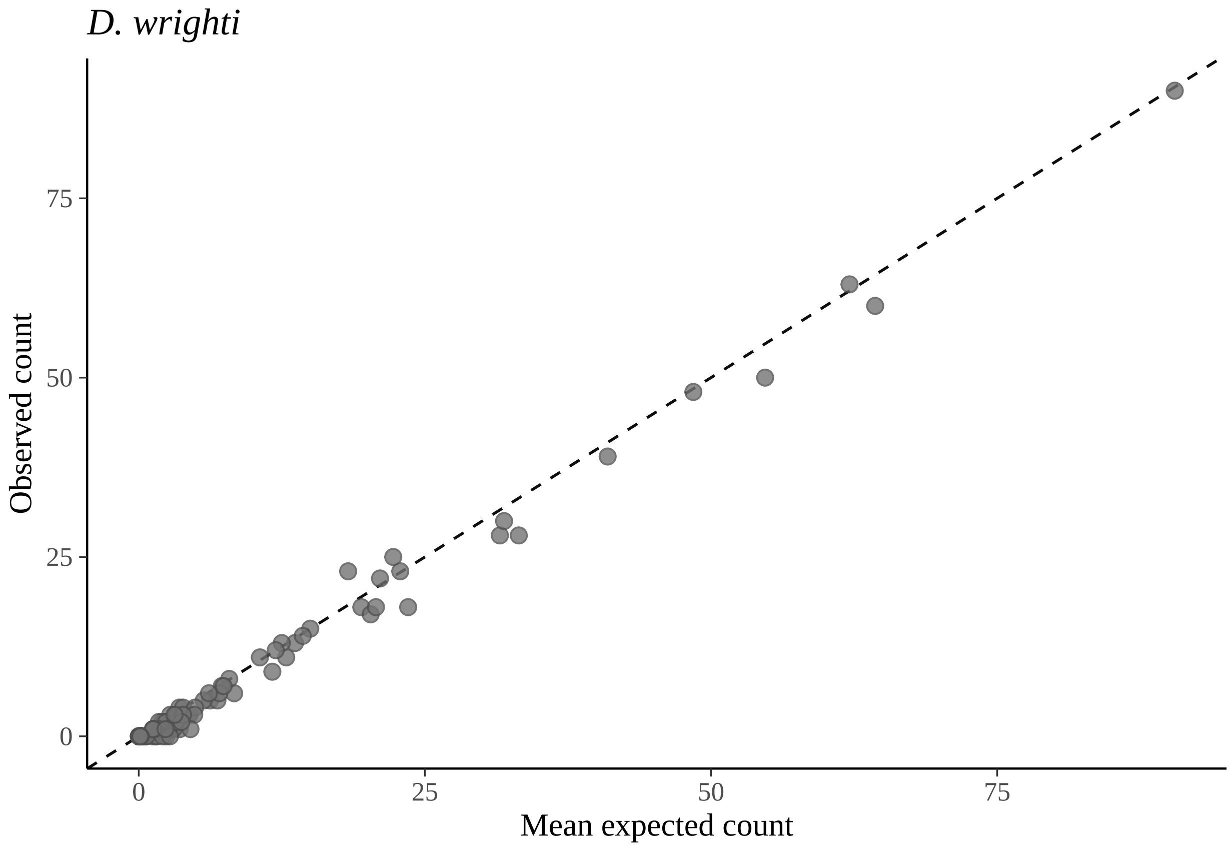

RMSE of posterior predictive

```
## [1] 1.315241
```

Posterior predictive check for each visit

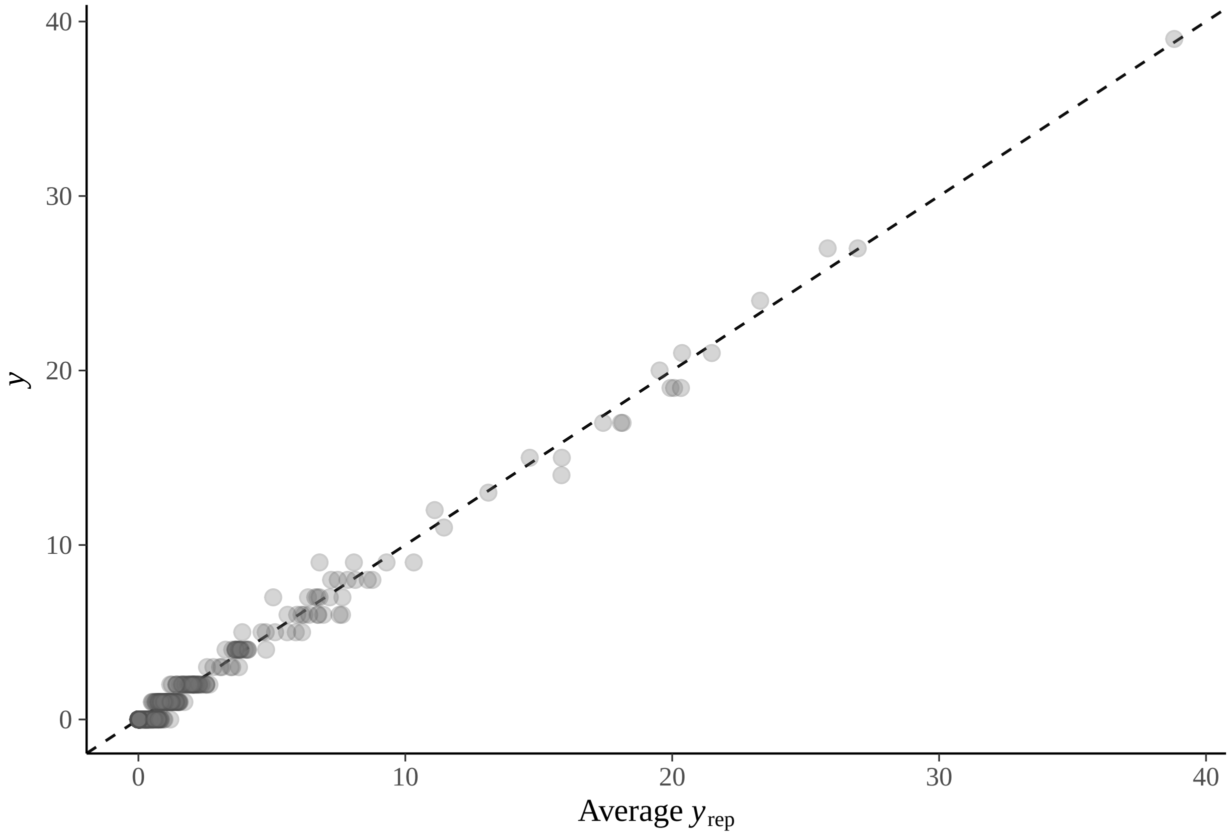

RMSE of posterior predictive for observations per visit

```
## [1] 0.7504749
```

RMSE of posterior predictive

```
## [1] 0.3356226
```

Eurycea wilderae

View Traceplots

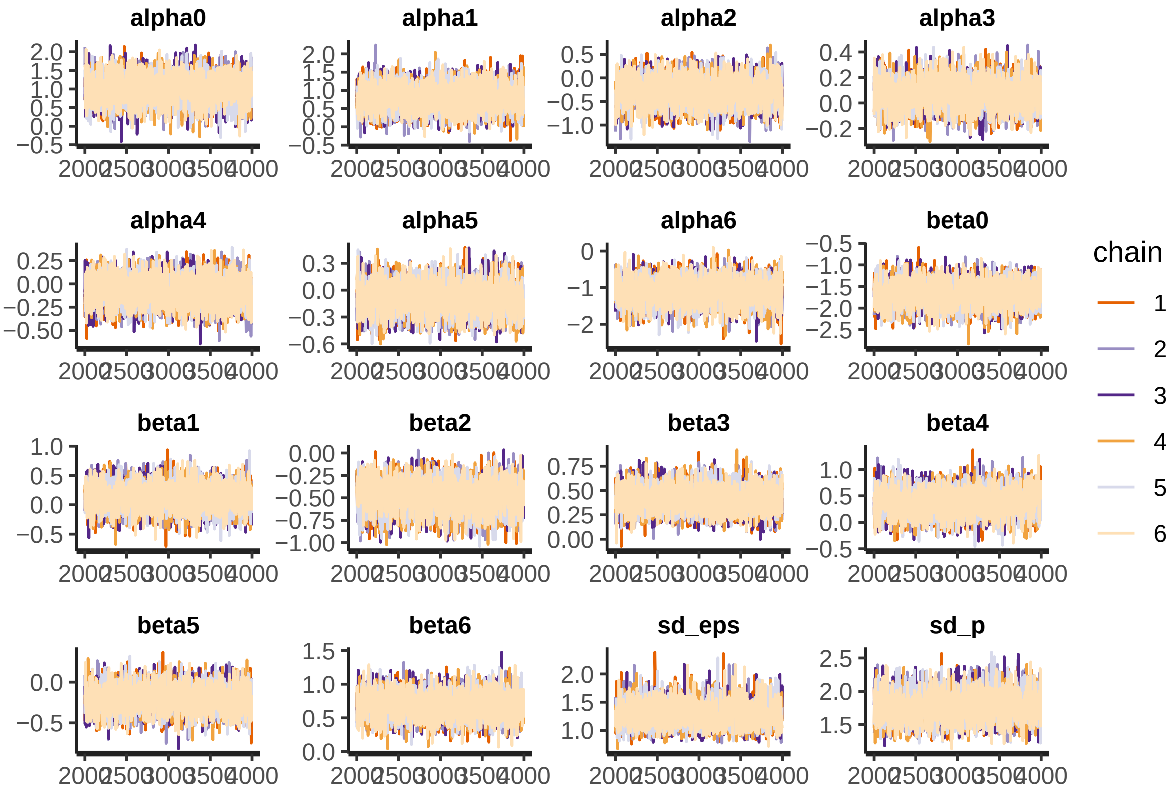

Check Domain Specific Expectations

Check N for Truncation

The augmentation to marginalize N out as a latent discrete requires setting an upper bound, K, to loop through. If K is too small the posterior will be truncated. Need to check for every N.

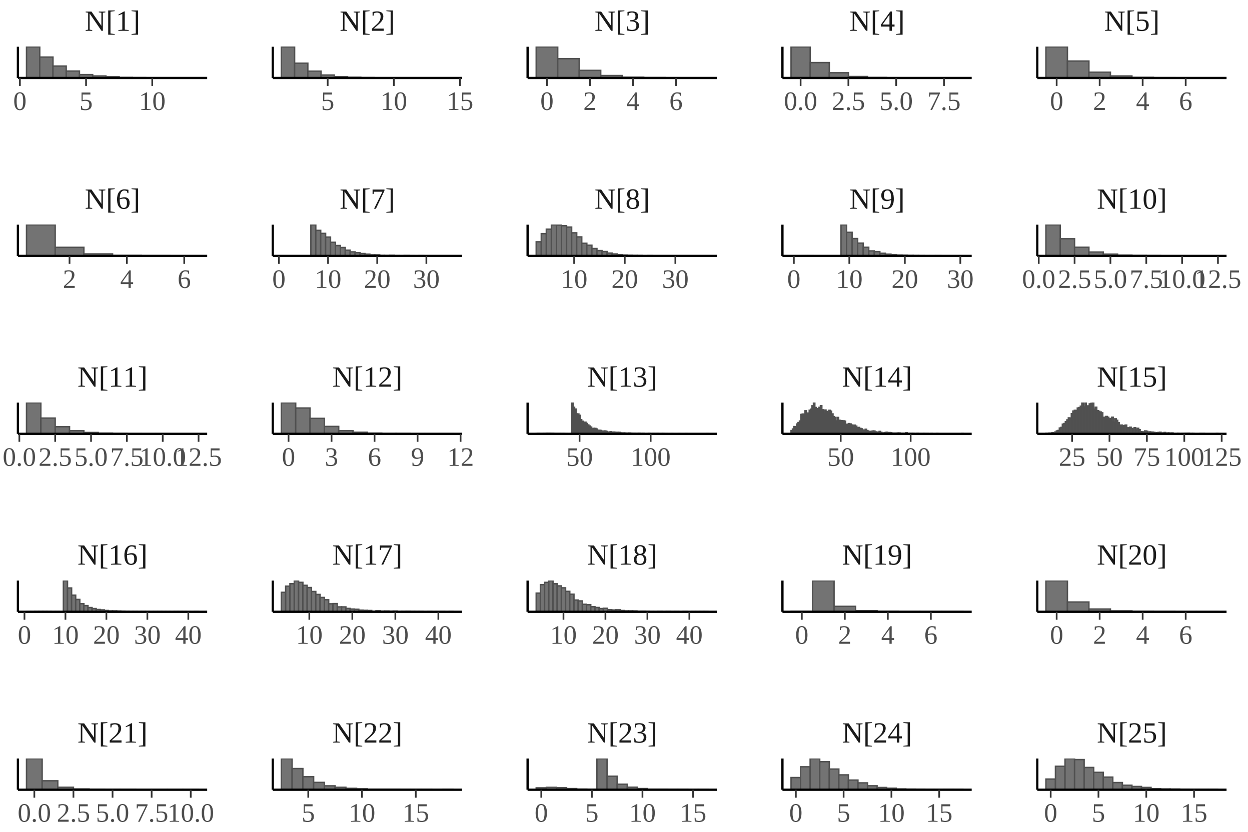
N[1]
0 5 10
N[2]
5 10 15
N[3]
0 2 4 6
N[4]
0.0 2.5 5.0 7.5
N[5]
0 2 4 6
N[6]
2 4 6
N[7]
0 10 20 30
N[8]
10 20 30
N[9]
0 10 20 30
N[10]
N[11]
N[12]
0 3 6 9 12
N[13]
50 100
N[14]
50 100
N[15]
N[16]
0 10 20 30 40
N[17]
10 20 30 40
N[18]
10 20 30 40
N[19]
0 2 4 6
N[20]
0 2 4 6
N[21]
N[22]
5 10 15
N[23]
0 5 10 15
N[24]
0 5 10 15
N[25]
0 5 10 15

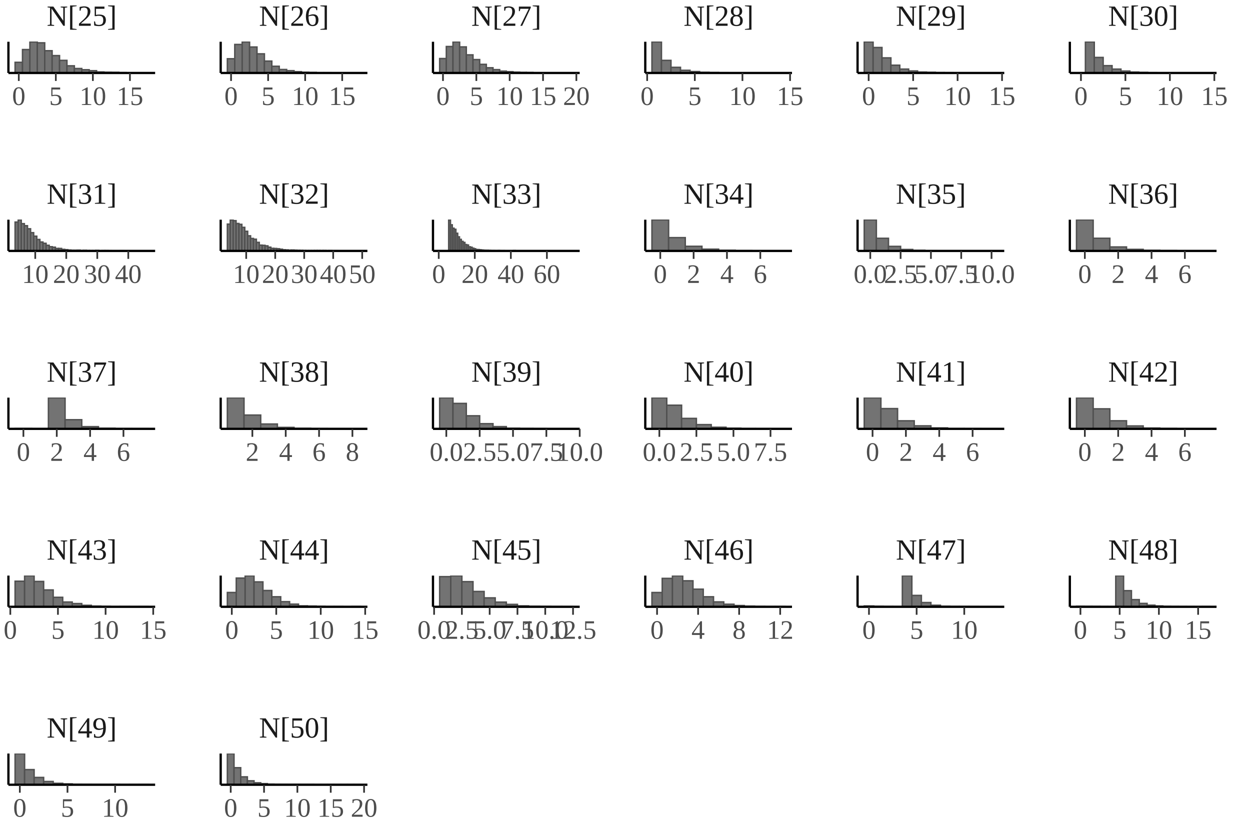
N[25]
N[26]
N[27]
N[28]
N[29]
N[30]
N[31]
N[32]
N[33]
N[34]
N[35]
N[36]
N[37]
N[38]
N[39]
N[40]
N[41]
N[42]
N[43]
N[44]
N[45]
N[46]
N[47]
N[48]
N[49]
N[50]

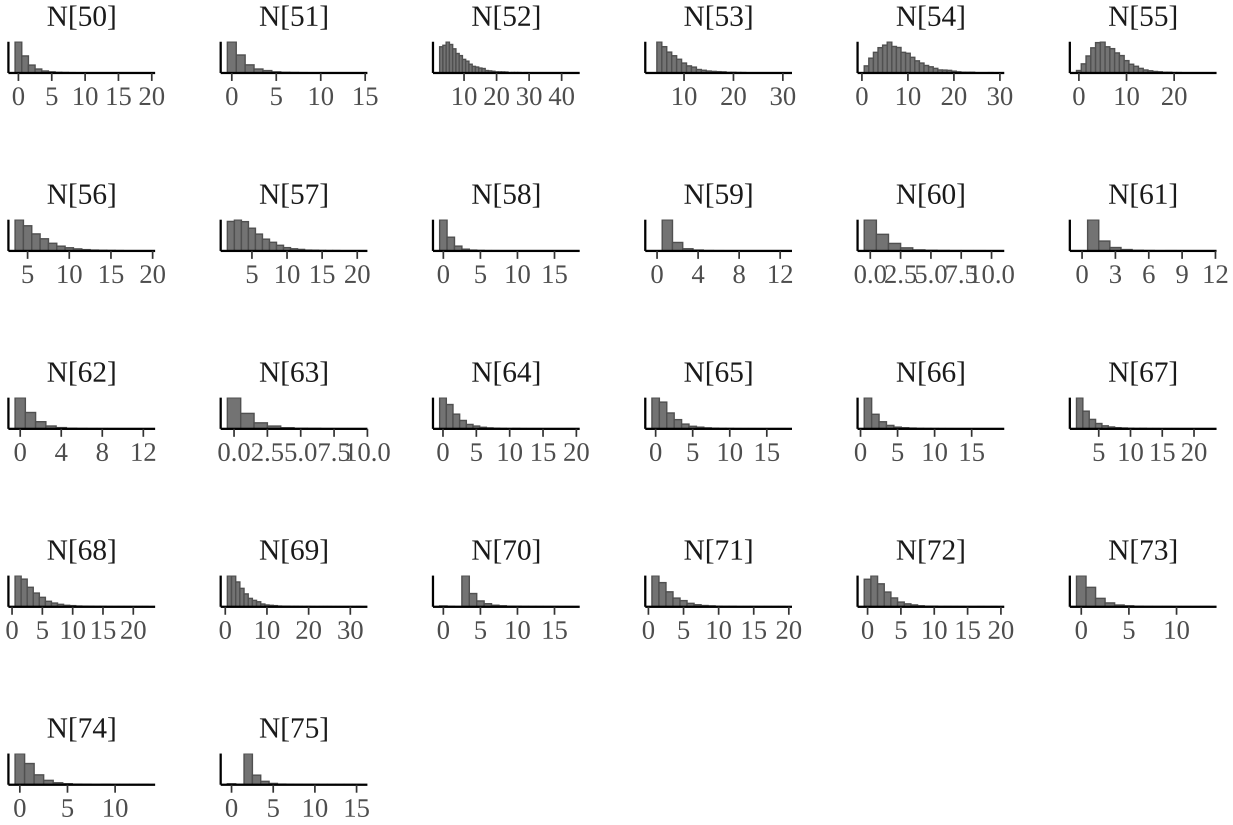
N[50]
0 5 10 15 20
N[51]
0 5 10 15
N[52]
10 20 30 40
N[53]
10 20 30
N[54]
0 10 20 30
N[55]
0 10 20
N[56]
5 10 15 20
N[57]
5 10 15 20
N[58]
0 5 10 15
N[59]
0 4 8 12
N[60]
N[61]
0 3 6 9 12
N[62]
0 4 8 12
N[63]
N[64]
0 5 10 15 20
N[65]
0 5 10 15
N[66]
0 5 10 15
N[67]
5 10 15 20
N[68]
0 5 10 15 20
N[69]
0 10 20 30
N[70]
0 5 10 15
N[71]
0 5 10 15 20
N[72]
0 5 10 15 20
N[73]
0 5 10
N[74]
0 5 10
N[75]
0 5 10 15

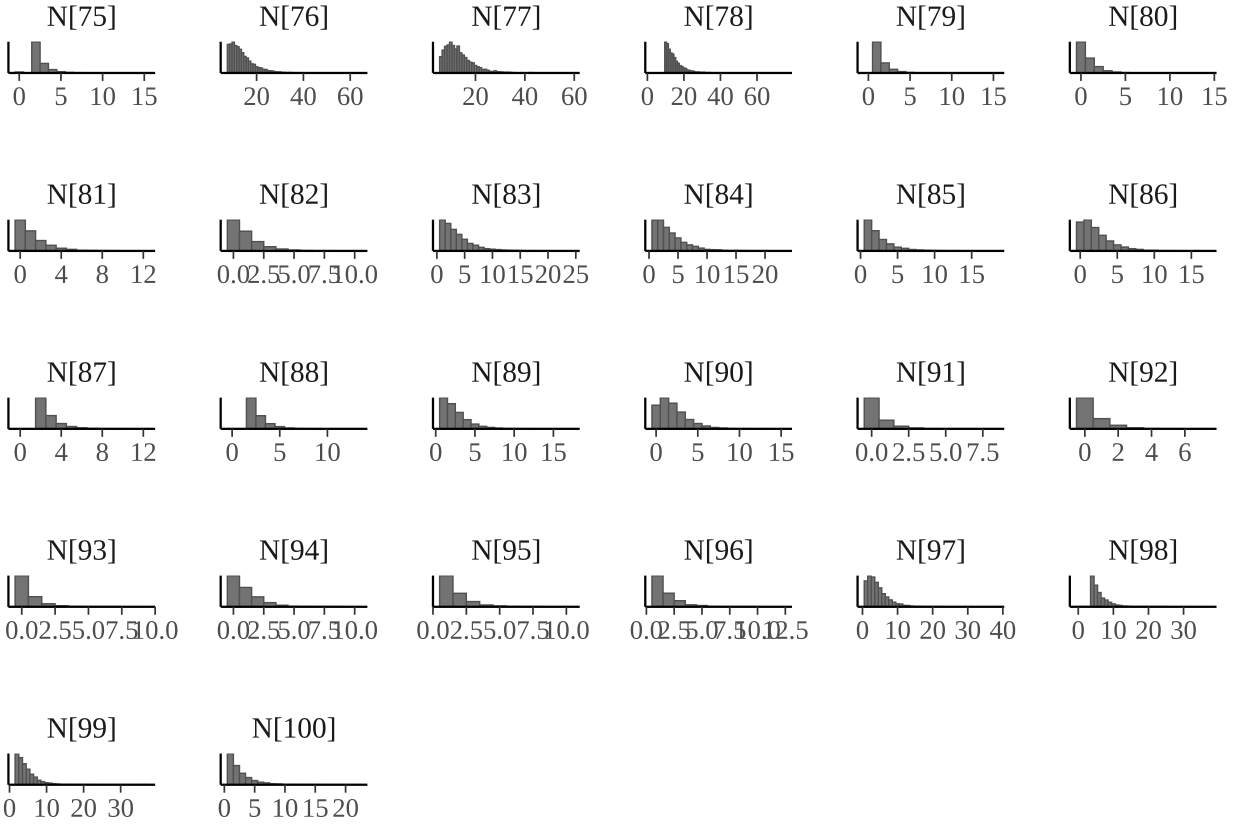
N[75]
N[76]
N[77]
N[78]
N[79]
N[80]
N[81]
N[82]
N[83]
N[84]
N[85]
N[86]
N[87]
N[88]
N[89]
N[90]
N[91]
N[92]
N[93]
N[94]
N[95]
N[96]
N[97]
N[98]
N[99]
N[100]

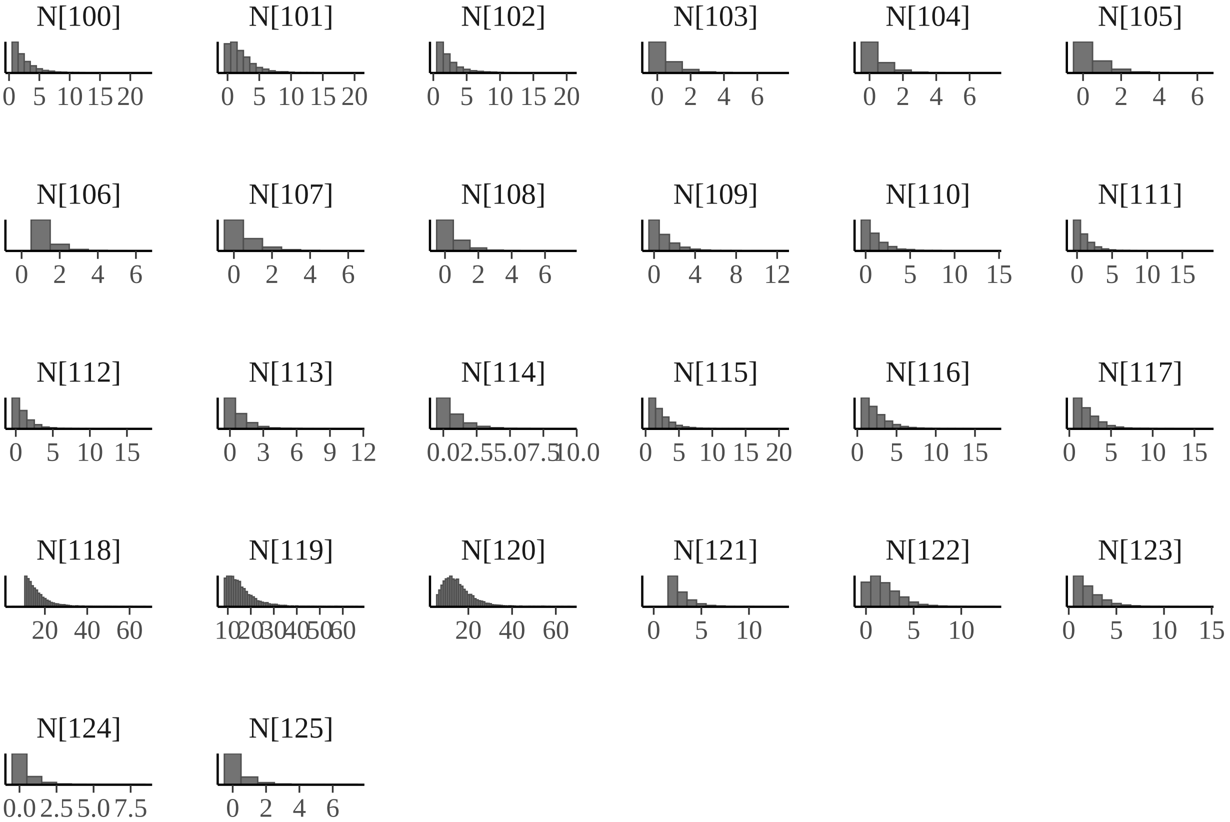
N[100]
0 5 10 15 20
N[101]
0 5 10 15 20
N[102]
0 5 10 15 20
N[103]
0 2 4 6
N[104]
0 2 4 6
N[105]
0 2 4 6
N[106]
0 2 4 6
N[107]
0 2 4 6
N[108]
0 2 4 6
N[109]
0 4 8 12
N[110]
0 5 10 15
N[111]
0 5 10 15
N[112]
0 5 10 15
N[113]
0 3 6 9 12
N[114]
0.0 2.5 5.0 7.5 10.0
N[115]
0 5 10 15 20
N[116]
0 5 10 15
N[117]
0 5 10 15
N[118]
20 40 60
N[119]
10 20 30 40 50 60
N[120]
20 40 60
N[121]
0 5 10
N[122]
0 5 10
N[123]
0 5 10 15
N[124]
0.0 2.5 5.0 7.5
N[125]
0 2 4 6

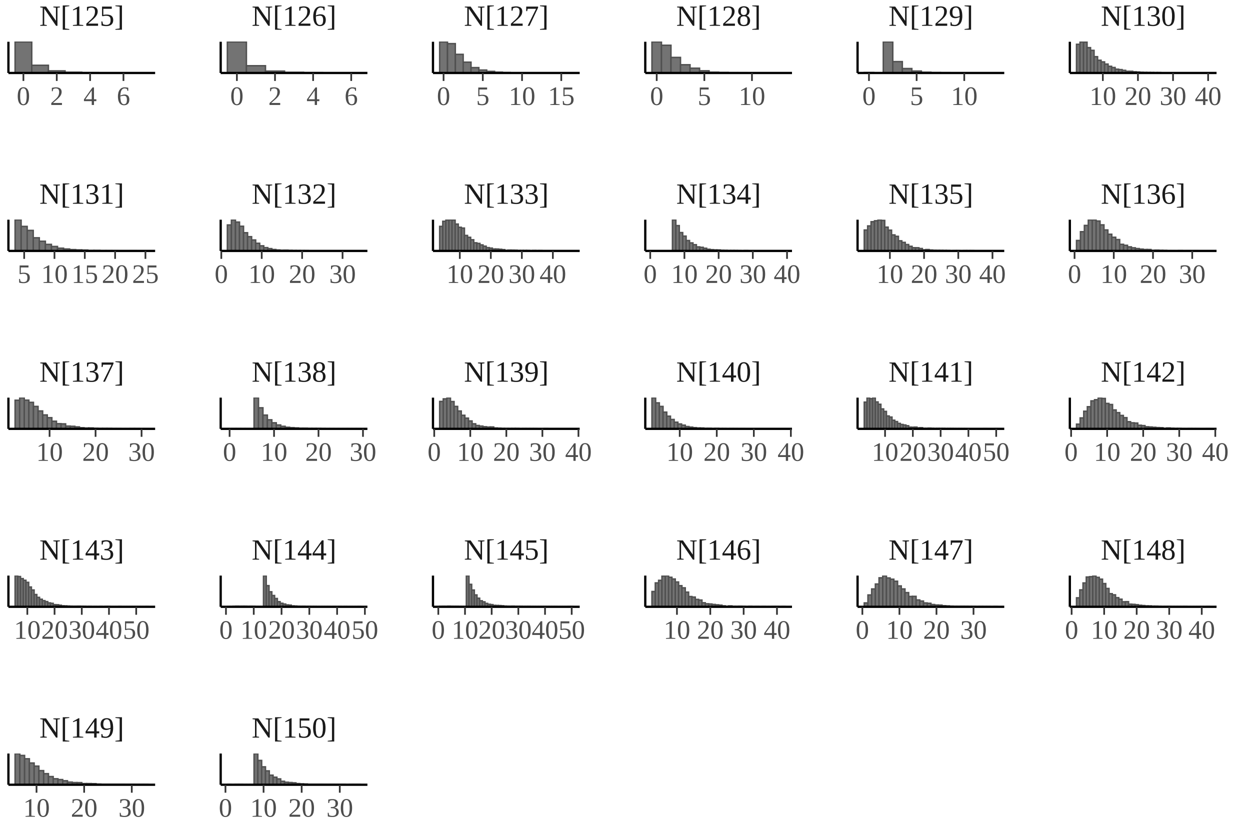
N[125]
0 2 4 6
N[126]
0 2 4 6
N[127]
0 5 10 15
N[128]
0 5 10
N[129]
0 5 10
N[130]
10 20 30 40
N[131]
5 10 15 20 25
N[132]
0 10 20 30
N[133]
10 20 30 40
N[134]
0 10 20 30 40
N[135]
10 20 30 40
N[136]
0 10 20 30
N[137]
10 20 30
N[138]
0 10 20 30
N[139]
0 10 20 30 40
N[140]
10 20 30 40
N[141]
1020304050
N[142]
0 10 20 30 40
N[143]
1020304050
N[144]
0 1020304050
N[145]
0 1020304050
N[146]
10 20 30 40
N[147]
0 10 20 30
N[148]
0 10 20 30 40
N[149]
10 20 30
N[150]
0 10 20 30

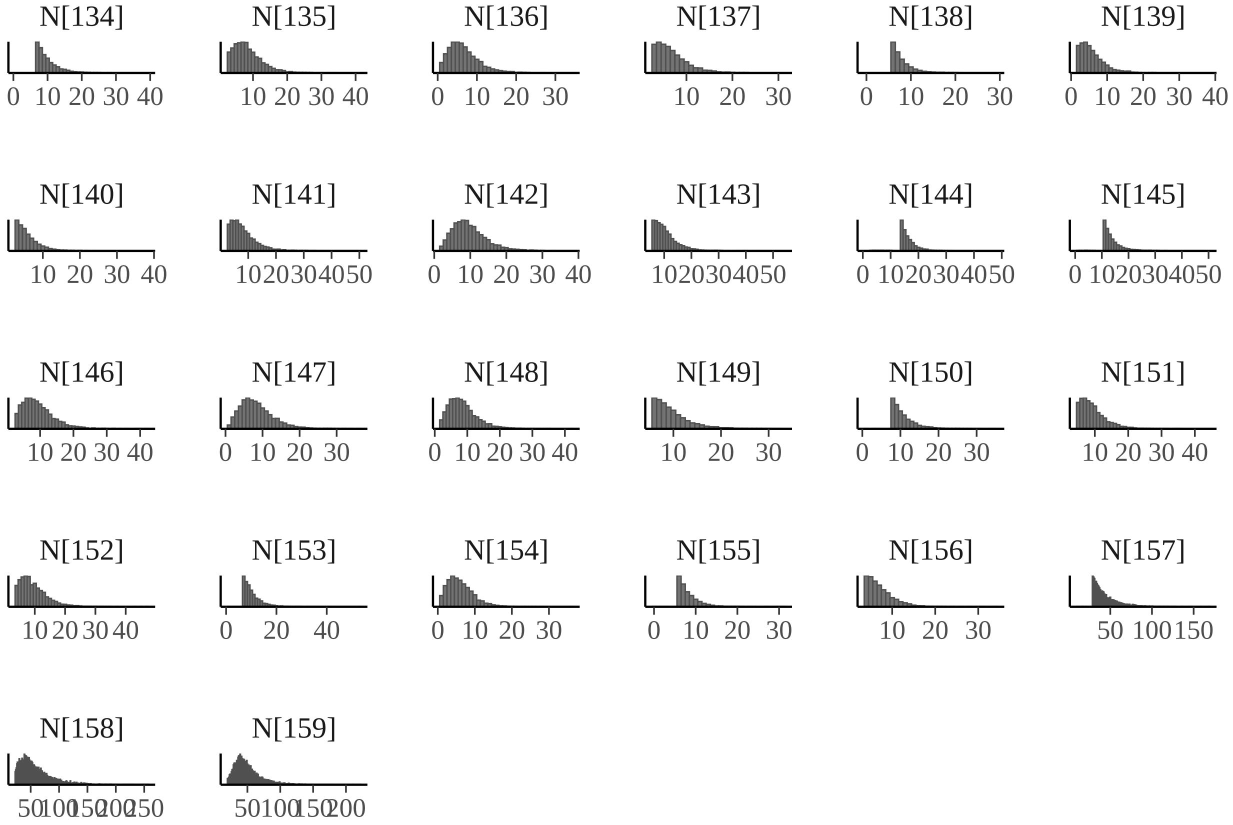


```
## null device
##           1
```

Check detection

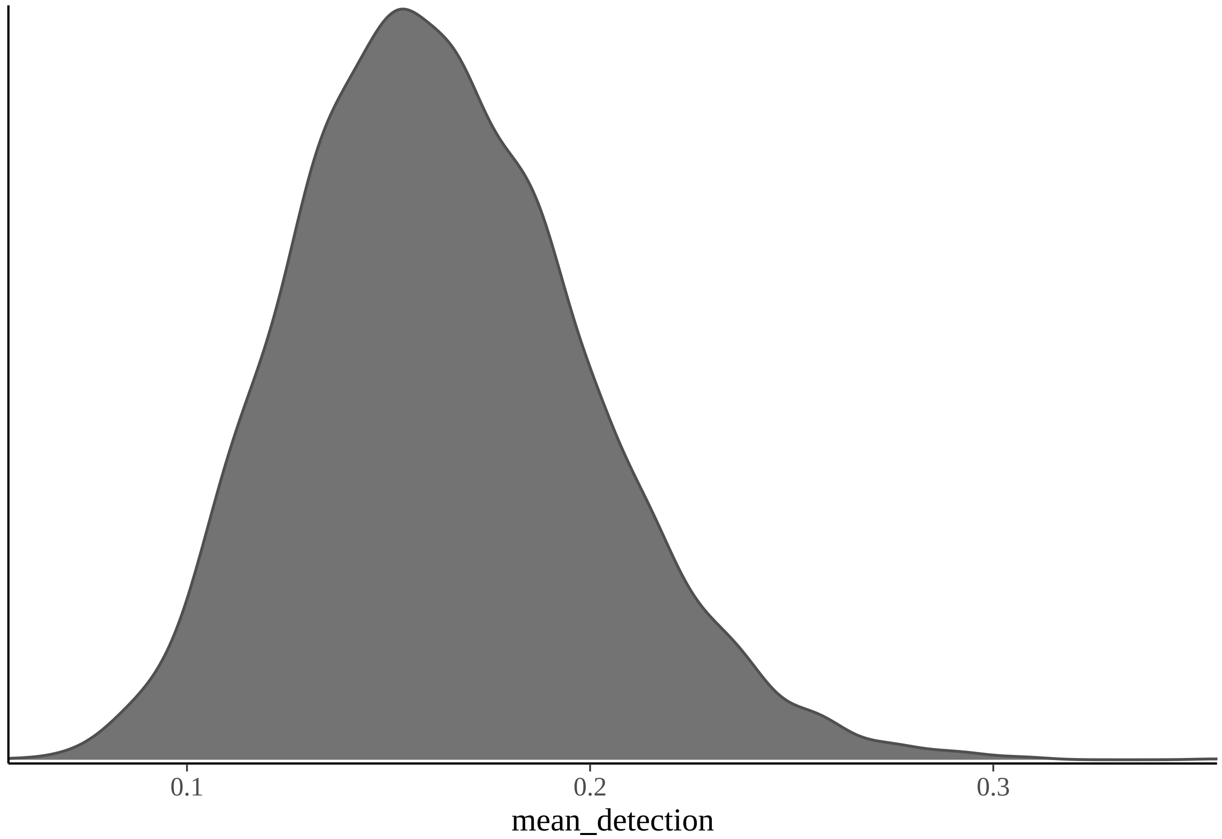

Check Divergences and Pairwise Correlations

```
## 0 of 6000 iterations ended with a divergence.
```

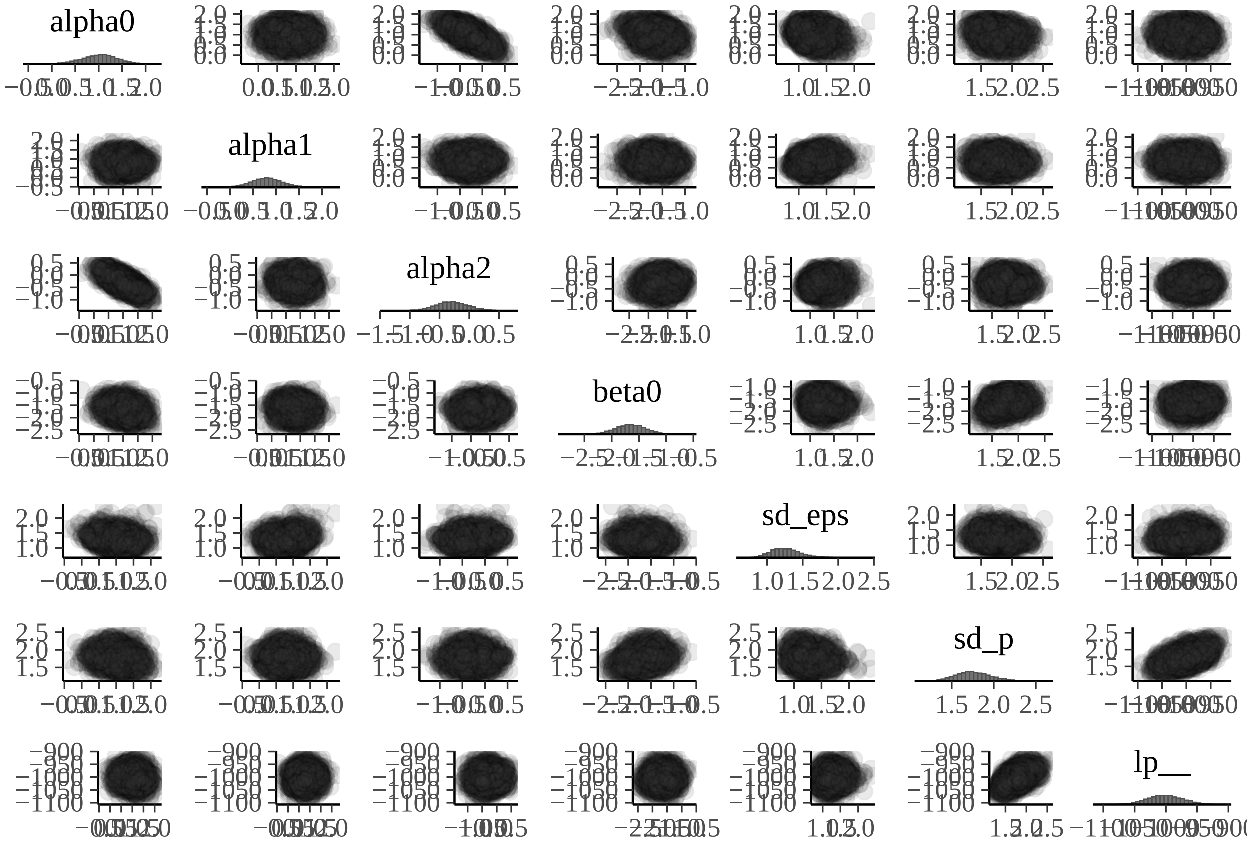

Check Energy and Treedepth

```
## E-BFMI indicated no pathological behavior.
```

```
## 0 of 6000 iterations saturated the maximum tree depth of 10.
```

Summarize Samples Sizes and Mixing

Effective samples sizes using rstan::monitor following Hoffman and Gelman (2014) to be more reliable and accurate (as in Monnahan et al. 2017) - UPDATE - now follow Vehtari et al. 2019.

effective sample sizes (should be > 100) - Aki Vehtari, Andrew Gelman, Daniel Simpson, Bob Carpenter, and Paul-Christian Bürkner (2019). Rank-normalization, folding, and localization: An improved R-hat for assessing convergence of MCMC. arXiv preprint arXiv:1903.08008.

```
## [1] 1.003496
```

```
## [1] 2495
```

```
## [1] 3415
```

Posterior Predictive Checks

Examine posterior prredictions of total counts across all 5 visits

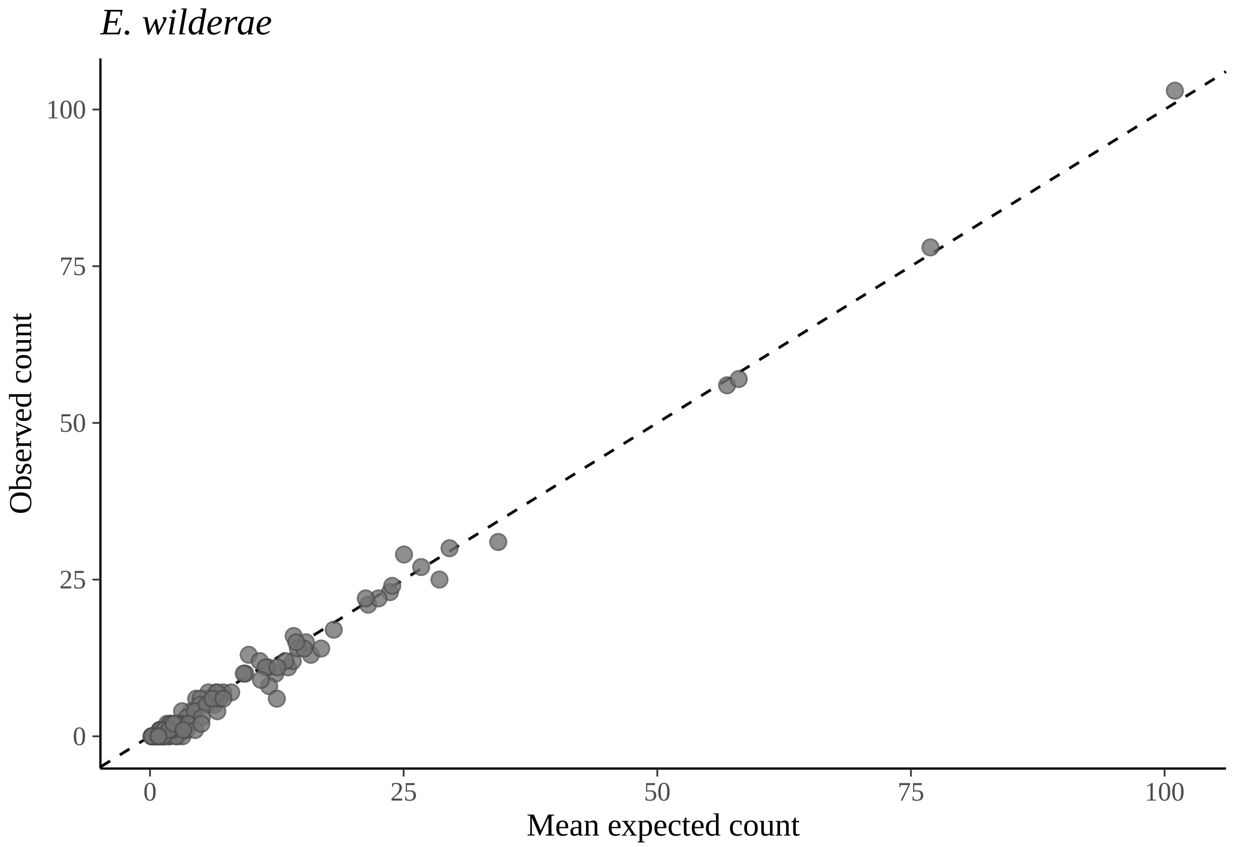

RMSE of posterior predictive

```
## [1] 1.404227
```

Posterior predictive check for each visit

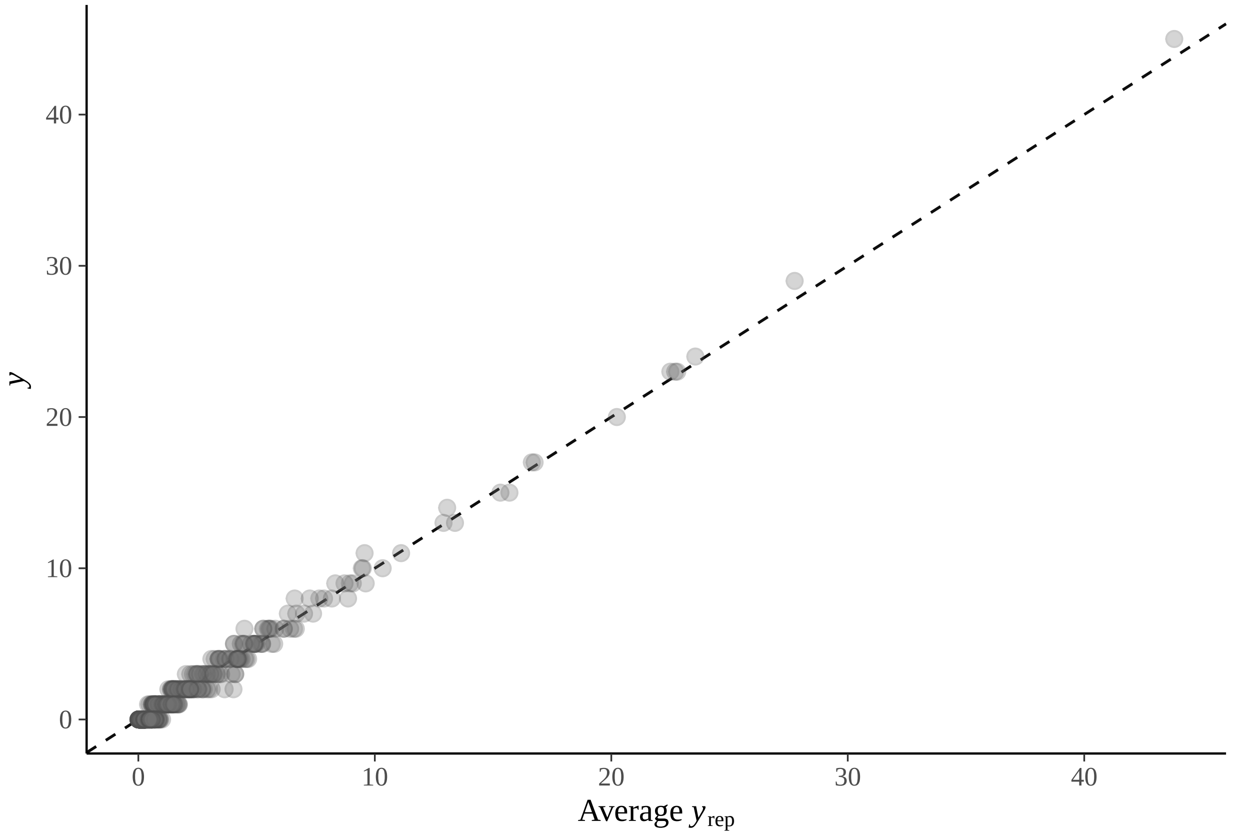

RMSE of posterior predictive for observations per visit

```
## [1] 0.8349771
```

RMSE of posterior predictive

```
## [1] 0.3734131
```
